# Cell-to-Cell Variation in Defective Virus Expression and Effects on Host Responses during Influenza Virus Infection

**DOI:** 10.1128/mBio.02880-19

**Published:** 2020-01-14

**Authors:** Chang Wang, Christian V. Forst, Tsui-wen Chou, Adam Geber, Minghui Wang, Wissam Hamou, Melissa Smith, Robert Sebra, Bin Zhang, Bin Zhou, Elodie Ghedin

**Affiliations:** a Center for Genomics and Systems Biology, Department of Biology, New York University, New York, New York, USA; b Mount Sinai Center for Transformative Disease Modeling, Department of Genetics and Genomic Sciences, Icahn Institute of Genomics and Multiscale Biology, Icahn School of Medicine at Mount Sinai, New York, New York, USA; c College of Global Public Health, New York University, New York, New York, USA; Vanderbilt University Medical Center

**Keywords:** influenza A virus, defective viral genome, host immune response, single-cell RNA-seq, viral transcription

## Abstract

Virus and host factors contribute to cell-to-cell variation in viral infections and determine the outcome of the overall infection. However, the extent of the variability at the single-cell level and how it impacts virus-host interactions at a system level are not well understood. To characterize the dynamics of viral transcription and host responses, we used single-cell RNA sequencing to quantify at multiple time points the host and viral transcriptomes of human A549 cells and primary bronchial epithelial cells infected with influenza A virus. We observed substantial variability in viral transcription between cells, including the accumulation of defective viral genomes (DVGs) that impact viral replication. We show (i) a correlation between DVGs and virus-induced variation of the host transcriptional program and (ii) an association between differential inductions of innate immune response genes and attenuated viral transcription in subpopulations of cells. These observations at the single-cell level improve our understanding of the complex virus-host interplay during influenza virus infection.

## Abstract (precis)

Defective influenza virus particles generated during viral replication carry incomplete viral genomes and can interfere with the replication of competent viruses. These defective genomes are thought to modulate the disease severity and pathogenicity of an influenza virus infection. Different defective viral genomes also introduce another source of variation across a heterogeneous cell population. Evaluating the impact of defective virus genomes on host cell responses cannot be fully resolved at the population level, requiring single-cell transcriptional profiling. Here, we characterized virus and host transcriptomes in individual influenza virus-infected cells, including those of defective viruses that arise during influenza A virus infection. We established an association between defective virus transcription and host responses and validated interfering and immunostimulatory functions of identified dominant defective viral genome species *in vitro*. This study demonstrates the intricate effects of defective viral genomes on host transcriptional responses and highlights the importance of capturing host-virus interactions at the single-cell level.

## INTRODUCTION

The productivity of viral replication at the cell population level is determined by cell-to-cell variation in viral infection (1). The genetically diverse nature of RNA viruses and the heterogeneity of host cell states contribute to this intercell variability, which can impact therapeutic applications (2, 3). Although previous studies of cell-to-cell variation during viral infection have centered mainly on nonsegmented viruses, such as poliovirus (2, 4), vesicular stomatitis virus (VSV) (5, 6), Sendai virus (SeV) (7, 8), dengue virus (DENV) (9), and zika virus (ZIKV) (10), heterogeneity across cells may be more complicated for viruses with a segmented genome, such as influenza A virus (IAV). IAV-infected cells display substantial cell-to-cell variation as it pertains to relative abundances of different viral genome segments (1, 11), transcripts (12–15), and their encoded proteins (16), which can result in nonproductive infection in a large fraction of cells.

Influenza virus defective interfering particles (DIs) readily generated during successive high multiplicities of infection (MOIs) in cell culture passages (17–21) and observed in natural infections (22, 23) contribute further to the observed cell-to-cell variation in infection. Influenza virus DIs have defective viral genomes (DVGs) that carry large internal deletions but retain conserved 5′ and 3′ termini identical to those in the full-length genomes (24, 25). They have a significant effect on productive infection (26), as they possess the ability to interfere with the replication of infectious viruses (18). Because DIs diminish the productivity of infectious progeny and may impact disease outcome, they are of great interest for therapeutic and prophylactic purposes (reviewed in references 27 to 30). A specific influenza virus DI (i.e., DI244) was shown to effectively provide protection against IAV infection in mice (31, 32) and ferrets (33, 34). One of the ways influenza virus DIs are thought to modulate viral infections is through interaction with a cytosolic pattern recognition receptor (PRR), retinoic acid-inducible gene I (RIG-I), essential for interferon (IFN) induction (35). It has been assumed that, besides having enhanced IFN induction *in vitro* (36–39) and *in vivo* (40), DIs may compete with standard viruses for cellular resources (reviewed in references 27 to 30 and 36). Recent studies on paramyxovirus revealed high heterogeneity in the accumulation of copy-back DVGs, resulting in the establishment of persistent infection in a subpopulation of cells (8) and differential levels of production of standard and defective viral particles (7). However, similar studies have not been done with influenza virus DVGs.

While diverse DIs can arise during IAV infection (40, 41), the emergence and accumulation of distinct DVGs and their impact on host gene expression have not been well characterized at the population level nor at a single-cell resolution. Using single-cell transcriptome sequencing (RNA-seq), which allows us to probe viral and host transcriptomes simultaneously in the same cells and determine the abundance and diversity of DVGs, we monitored host-virus interactions in cultured cells over the course of IAV infection. These data established a temporal association between the level of viral transcription and effects on the host transcriptome and characterized the diversity and accumulation of DVG transcripts.

## RESULTS

### Cell-to-cell variation in virus gene expression.

To determine how both the viral and host cell transcriptional programs relate to each other over the course of an influenza virus infection, we (i) infected two cell types, the adenocarcinomic human alveolar basal epithelial A549 cell line and human bronchial epithelial cells (HBEpC), at a high multiplicity of infection (MOI; 5) with A/Puerto Rico/8/34 (H1N1) (PR8) and (ii) performed transcriptome profiling by conventional bulk RNA-seq and a droplet-based single-cell RNA-seq approach. A high-MOI infection ensures that virtually all the cells can rapidly be infected, promotes the accumulation of DVGs, and consequently enables the characterization of the host response and DVG diversity. We first determined the percentage of reads that uniquely aligned with viral genes from the total number of mapped reads to obtain the relative abundances of virus transcripts within cells at each time point. Similarly to what has been observed at early stages of infection during a low-MOI infection with IAV (12), the relative abundances of virus transcripts were heterogeneous across cells from both cell types, with 0 to 70% of the total reads in each cell being derived from virus transcripts and the relative abundances of these transcripts increasing over time (see Fig. S1a in the supplemental material). The same trend was also seen when we analyzed segment-specific virus transcripts within individual cells over the course of the infection (Fig. S1b).

10.1128/mBio.02880-19.1 FIG S1 Distribution of the relative abundances of virus transcripts within individual cells over the course of the infection. (a) The overall relative abundance of virus transcripts was calculated as the percentage of viral reads in the pool of all the reads for each cell. The dots are colored by the time points. All pairwise comparisons were performed with the one-tailed Wilcoxon rank sum test, for which the null hypothesis was that cells harvested at an earlier time point have a lower median relative abundance of virus transcripts than those harvested at a later time point. Asterisks over the boxes denote the statistical significance levels of pairwise comparisons: *, *P* ≤ 0.05; **, *P* ≤ 0.01; ***, *P* ≤ 0.001; and ****, *P* ≤ 0.0001. ns, not significant. The data for HBEpC at 12 hpi were collected from a repeated infection assay, due to the initial failure of single-cell library preparation for the corresponding sample. (b) Distribution of the relative abundances of virus transcripts derived from each segment within individual cells over the course of the infection. All box plots show the first and third quantiles as the lower and upper hinges, the median in the center, and a 1.5 interquartile range (IQR) from the first and third quantiles as the whiskers. Download FIG S1, PDF file, 0.2 MB. Copyright © 2020 Wang et al. 2020 Wang et al. This content is distributed under the terms of the Creative Commons Attribution 4.0 International license.

### Heterogeneity of defective virus segment expression across the cell population.

**FIG 1:**
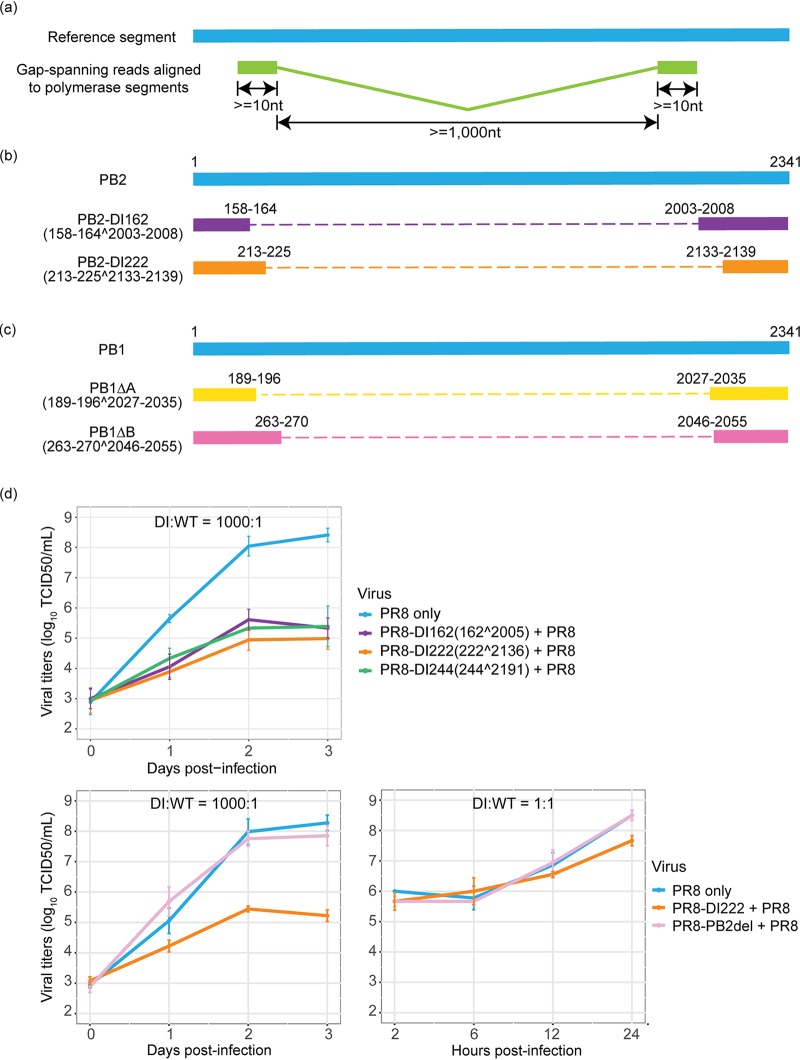
Characterization of DVG transcripts and validation of interfering capacity. (a) Selection criteria for reads considered to be representatives of DVGs. Given the lengths of the reads, we required that each portion of the gap-spanning reads were at least 10 bp in length. Considering that DVGs typically lose approximately 80% of their original sequences and considering the distribution in the size of the deletions detected in this study, we also required that the size of the gap should be at least 1 kb. (b and c) Schematic diagram showing two types of defective virus transcripts derived from the PB2 (b) and PB1 (c) segments, respectively. The caret (^) denotes a deletion event, and the numbers before and following it indicate the coordinates of the junction sites. (d) Interfering capacity of generated defective interfering particles (DIs) carrying identified deletion sites in the PB2 segment during coinfection with the WT PR8 virus. A549 cells were coinfected with WT virus and one type of DI at a ratio of 1:1,000 or 1:1. Viral titers were measured by the TCID_50_ assay on MDCK cells. Four types of defective viruses, including two DIs identified in this study, a previously reported DI244 virus (31) that has been considered a potential candidate for antiviral therapy (27), and a PB2 polymerase-defective PR8 virus mutant (i.e., PB2del) that carries 1 nucleotide deletion in the coding sequence of the PB2 gene, were tested. For each type of DI, the information about the junction sites is given in parentheses. The error bar representing the standard deviation of the mean was obtained from three biological replicates. Each panel shows the results of an independent interference assay.

Since DVGs are known to accumulate in cell culture, especially during high-MOI infections (17–21), and can serve as templates for transcription (42), we characterized their abundances and diversity by examining gap-spanning reads in the sequencing data. To determine the relative abundances of DVGs identified, we calculated the frequency of each unique DVG transcript type (determined by 5′/3′ gap coordinates) across single cells and the ratio of these to non-gap-spanning transcripts derived from the corresponding viral segments (i.e., DVG/full-length genome [FL] ratio). Although DVG transcripts could be detected in all viral segments, those originating from the polymerase segments (i.e., PB2, PB1, and PA) occurred with the highest abundances and frequency in both cell types over the course of the infection (data available on GitHub, https://github.com/GhedinLab/Single-Cell-IAV-infection-in-monolayer/blob/master/AdditionalFiles/AdditionalFile1_DVG-from-all-segments_DVG-FL-ratios.pdf), consistent with observations made in other studies (22, 43). We thus focused our analyses on the detection of DVG transcripts derived from those segments. We collected reads with large internal deletions spanning ≥1,000 nucleotides (nt) (Fig. 1a) and identified the junction coordinates of these gap-spanning reads. We observed a diverse pool of DVG transcripts, including some shared with the viral RNA (vRNA) segments from the PR8 stock (data are available on GitHub, https://github.com/GhedinLab/Single-Cell-IAV-infection-in-monolayer/blob/master/AdditionalFiles/AdditionalFile2_Polymerase-segments-DVG-species.pdf). The sizes for the majority of these DVG transcripts are estimated to be between 300 nt and 1,000 nt. To evaluate the accumulation of DVG transcripts in the virus transcript pool over the course of the infection, we compared changes in the ratios of DVG transcripts to FL transcripts derived from a given polymerase segment (DVG/FL) across time points. The polymerase DVG/FL ratios increased significantly between the early (6 h postinfection [hpi]) and late (24 hpi) stages of the infection (*P* < 2.2 × 10^−16^) and displayed a high level of heterogeneity across single cells from both cell types (Fig. S2).

10.1128/mBio.02880-19.2 FIG S2 Distribution of the DVG/FL ratios in A549 cells and HBEpC over the course of the infection. All pairwise comparisons were performed with the two-tailed Wilcoxon rank sum test, for which the null hypothesis was that cells harvested at two different time points have different median relative abundances of virus transcripts. Asterisks over the boxes denote the statistical significance levels of pairwise comparisons: *, *P* ≤ 0.05, **; *P* ≤ 0.01; ***, *P* ≤ 0.001; and ****, *P* ≤ 0.0001. ns, not significant. All box plots show the first and third quantiles as the lower and upper hinges, the median in the center, and a 1.5 interquartile range (IQR) from the first and third quantiles as the whiskers. Download FIG S2, PDF file, 0.1 MB. Copyright © 2020 Wang et al. 2020 Wang et al. This content is distributed under the terms of the Creative Commons Attribution 4.0 International license.

Interestingly, the junction sites aggregated in specific genomic regions, as they systematically occurred in a high percentage of cells. We identified two predominant types of DVG transcripts corresponding to defective PB2 and PB1 segments (Fig. 1b and c and Fig. S3 and S4). These same DVG transcripts were present in the stock, increased in prevalence over the course of the infection, and were conserved in different cell types and at different MOIs (data are available on GitHub, https://github.com/GhedinLab/Single-Cell-IAV-infection-in-monolayer/blob/master/AdditionalFiles/AdditionalFile3_Polymerase-DVG-species-abundance_previous-PB2-PB1.pdf), indicating likely carryover from the stock virus rather than *de novo* formation in each cell type. For PA, one particular DVG type that was present in the stock was found in A549 and Madin-Darby canine kidney (MDCK) cells at different MOIs, but not in HBEpC. Its prevalence was also much lower than for PB2 and PB1 DVGs (data are available on GitHub, https://github.com/GhedinLab/Single-Cell-IAV-infection-in-monolayer/blob/master/AdditionalFiles/AdditionalFile4_Polymerase-DVG-species-abundance_previous-data-and-PA-data.pdf). The dominant DVG PB2 and PB1 transcripts derived from corresponding defective segments in both the stock and the infected cells showed stable relative abundances across individual cells over the course of the infection, suggesting the persistence of the defective viral segments.

10.1128/mBio.02880-19.3 FIG S3 Percentages of A549 cells and HBEpC in which a given PB2-DVG transcript was detected versus the median of the DVG/FL ratio for that transcript in those cells over the course of the infection. Each type of PB2-DVG transcript is represented by a dot or triangle. A dot denotes the DVG transcripts also seen in the virus stock and a filled triangle denoted the *de novo*-generated transcripts. Two dominant PB2-DVG species are highlighted in slate blue and orange. Download FIG S3, PDF file, 0.9 MB. Copyright © 2020 Wang et al. 2020 Wang et al. This content is distributed under the terms of the Creative Commons Attribution 4.0 International license.

To determine if any of the inferred defective polymerase segments could lead to the generation of defective interfering particles (DIs), we tested their interference potential experimentally at the bulk level. We generated clonal PB8-DI162 and PR8-DI222 viruses carrying the deletion sites in the PB2 segment identified as those in two dominant PB2-DVG species and then coinfected each of the DIs with wild-type (WT) PR8 virus in A549 cells. Both the PR8-DI162 and PR8-DI222 viruses inhibited the productivity of WT virus as well as PR8-DI244, known to be an effective DI (31) (Fig. 1d). To confirm that inhibition is specific to the DIs and not due to competition between coinfecting strains, we also tested a full-length PR8 virus with a frame-shifting point mutation in the PB2 gene. Coinfection with this mutant virus did not affect the productivity of WT virus (Fig. 1d). The PR8-DI222 virus had a strong inhibitory effect at a DI/WT ratio of 1,000:1, and a mild effect could still be detected at a DI/WT ratio of 1:1 (Fig. 1d). This confirmed the interfering ability of the two types of defective PB2 segments identified in the single-cell data. The interference test was not intended to mimic a single-cell scenario, given the difficulties in recreating single-cell infection conditions at a bulk level.

### Cell-to-cell variation in concurrent virus-host transcriptional changes over the course of the infection.

**FIG 2:**
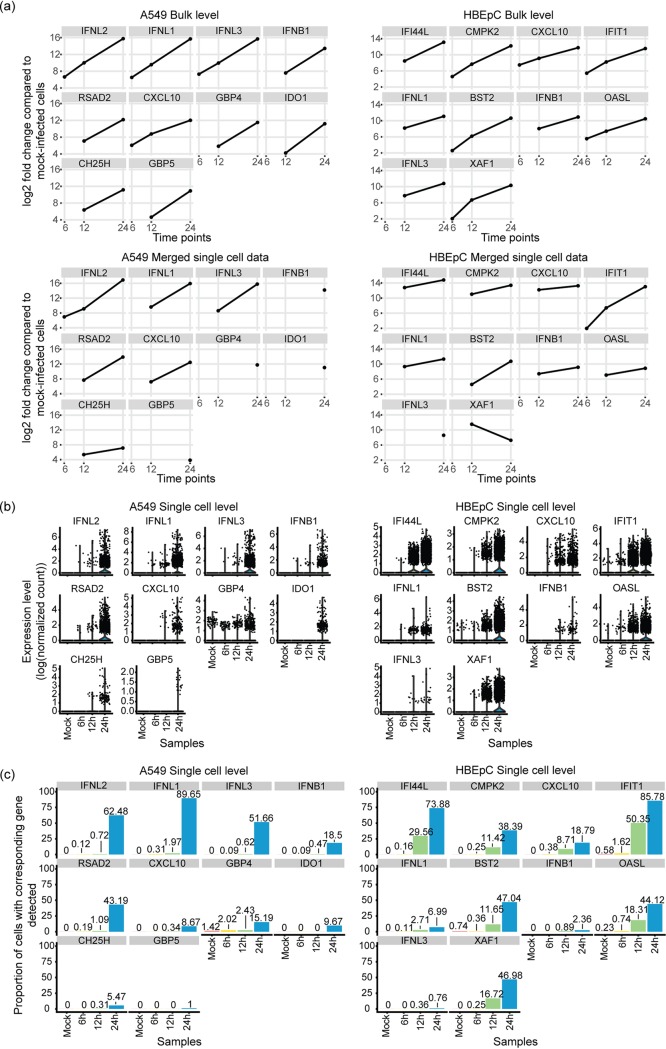
Comparison of the induction of interferons (IFNs) and interferon-stimulated genes (ISGs) at the bulk and single-cell levels. (a) Log_2_ fold changes of the top 10 differentially expressed genes with the greatest fold change by 24 hpi, including type I and III IFNs and some ISGs, compared to their expression in mock-infected cells detected in A549 cells and HBEpC over the course of the infection at the bulk level and their log_2_ fold changes in merged single-cell data, which mimic the bulk-level measurements. The results from edgeR are shown. (b) Expression of these genes at the single-cell level over the course of the infection in two cell types. Expression was calculated as log-transformed UMI counts normalized by the host library size. (c) Proportion of cells in which these genes are detected over the course of the infection in two cell types. The colors are consistent with those in panel b.

Viral infection can trigger massive changes in the host transcriptional program, including the activation of the interferon (IFN) response (reviewed in reference 44). Since the induction of IFNs is also subject to cell-to-cell variation (13, 15, 45), we first evaluated the expression frequency of type I IFN (e.g., beta IFN [IFN-β]) and type III IFN (i.e., IFN-λ) to determine the extent of the variation during infection. While these were significantly differentially expressed over the course of the infection at the population level, as measured by bulk RNA-seq (Fig. 2a), the expression of IFNs was detected in only less than 3% of cells until the late stage of infection, although this proportion increased over time (Fig. 2b and c). The same expression profile was observed for a number of IFN-stimulated genes (ISGs), such as *RSAD2*, *CXCL10*, *GBP4*, *GBP5*, *IDO1*, and *CH25H*, in A549 cells and for *IFI44L*, *CMPK2*, *IFIT1*, *BST2*, *OASL*, and *XAF1* in HBEpC (Fig. 2). *In silico* pooling of the single-cell data to mimic the population-level measurement resembles the bulk RNA-seq data, thus excluding the possibility of substantial technical limitation of single-cell RNA-seq (Fig. 2a).

**FIG 3:**
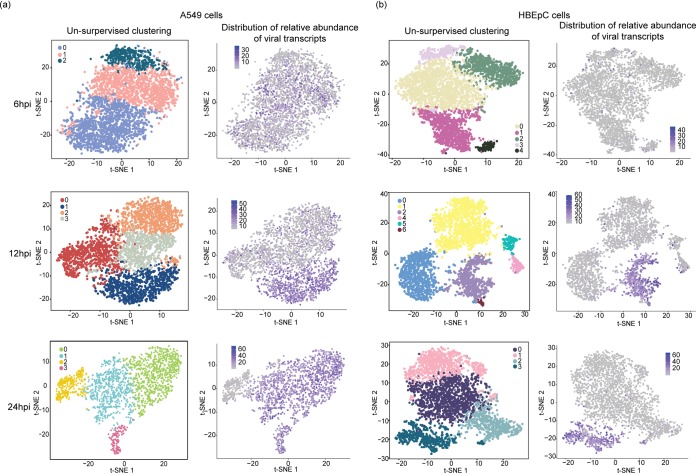
Comparison of cell clustering patterns and the distribution of virus transcript relative abundances. PR8 virus-infected A549 cells (a) and HBEpC (b) harvested at each time point were subjected to unsupervised clustering based on the host transcriptome and visualized on a *t*-SNE plot. Each dot on a *t*-SNE plot represents a cell. Cell clustering patterns, in which cells were colored by their cluster identity, are shown in the left panels, and the distribution of the relative abundances of virus transcripts at different time points in individual cells is shown in the right panels.

To evaluate cell-to-cell variation in the host response manifested by the alteration in the host transcriptome following IAV infection, we performed unsupervised cell clustering using solely the host transcriptional data. We then annotated single cells in each subpopulation with information about the relative abundances of virus transcripts and the DVG/FL ratio. We observed a correlative pattern between the distribution of the relative abundances of virus transcripts and the host transcriptional landscape across cells within a population, as cells with a high abundance of virus transcripts formed a separate cluster by 12 hpi in both cell types. This population structure, marked by a drastic difference in the level of viral transcription between clusters, was retained at 24 hpi (Fig. 3a and b). However, cell cycle status, which contributes to the heterogeneity of the cell population by 12 hpi and the formation of clusters in distinct cell cycle stages (Fig. S5a and Fig. S5b) with similar abundances of virus transcripts, does not seem to have a significant impact on population structure in A549 cells at 24 hpi. Consistently with previous reports on G_0_/G_1_ cell cycle arrest (46, 47), at 24 hpi, we observed a drastic increase in the proportion of cells in the G_0_/G_1_ phase compared to proportions at earlier time points (Fig. S5c). Interestingly, this same pattern was not observed in HBEpC at the same time point; in these cells, the host and viral transcriptome landscape at 24 hpi is similar to that in A549 cells at 12 hpi, suggesting a temporal difference in host response and viral replication between cell types.

10.1128/mBio.02880-19.4 FIG S4 Percentages of A549 cells and HBEpC in which a given DVG PB1 transcript was detected versus the median of the DVG/FL ratio for that transcript in those cells over the course of the infection. Two dominant DVG PB1 species are highlighted in yellow and pink. Download FIG S4, PDF file, 0.9 MB. Copyright © 2020 Wang et al. 2020 Wang et al. This content is distributed under the terms of the Creative Commons Attribution 4.0 International license.

10.1128/mBio.02880-19.5 FIG S5 Visualization of unsupervised cell clustering on a *t*-SNE plot for mock-infected A549 cells (a) and HBEpC (b) and the distribution of cell cycle genes within the population at each time point in two cell types. (a and b) Distribution of the cell cycle stage for each cell represented by a dot on the *t*-SNE plot. Dots were colored by either the cluster identities for the mock-infected cells or the cell cycle stages for mock- and PR8-infected cells. The cell cycle stage was assigned to each cell based on the expression level of a list of cell cycle markers. (c) Distribution of the percentages of cells in each cell cycle stage in two cell types. Download FIG S5, PDF file, 1.4 MB. Copyright © 2020 Wang et al. 2020 Wang et al. This content is distributed under the terms of the Creative Commons Attribution 4.0 International license.

**FIG 4:**
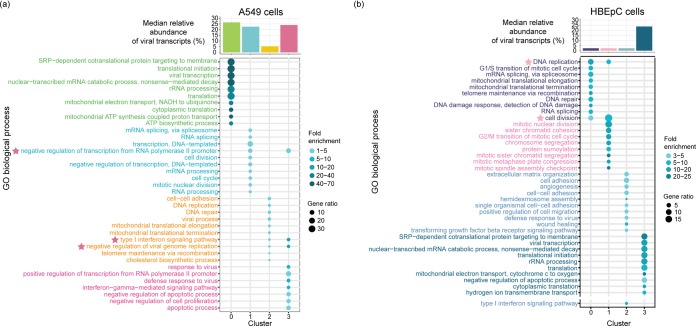
Overrepresented biological process gene ontology (GO) terms in A549 cells and HBEpC at 24 hpi. (a and b) Biological process GO terms associated with highly expressed genes in each cluster of A549 cells (a) and HBEpC (b). The top 10 overrepresented GO terms in each cluster are shown in the bubble charts. We also indicate another overrepresented GO term related to the type I IFN signaling pathway in cluster 2 of HBEpC, as shown separately in a lower section of the bubble chart. Each GO term is denoted by a bubble. The color intensity of each bubble indicates the fold enrichment of the corresponding GO term, and the size corresponds to the ratio of queried genes in the gene set associated with a given GO term. The GO terms enriched in each cluster are color-coded by cluster. The asterisks next to the GO terms in the bubble charts denote the GO terms enriched in two clusters, and their colors indicate the identity of the other cluster besides the one denoted by the color of the GO term. The bar chart above the bubble chart of the GO terms shows the median relative abundances of the virus transcripts across cells in each cluster. The colors of the bar are consistent with those of the GO terms.

To characterize host genes driving the changes associated with viral transcription, we assessed the overrepresentation of gene ontology (GO) terms associated with differentially expressed genes in each subpopulation of A549 cells and HBEpC compared to the other subpopulations of cells at 24 hpi. In two subpopulations of A549 cells with high relative abundances of virus transcripts (clusters 0 and 1 in Fig. 4a), there is an enrichment of genes involved in viral transcription, RNA processing, translation, signal recognition particle-dependent cotranslational protein targeting to the membrane, and mitochondrial electron transport (Fig. 4a). These GO terms are also associated with genes highly expressed in HBEpC, which have the highest abundances of virus transcripts at 24 hpi (cluster 3 in Fig. 4b) (*P* is <2.2 × 10^−16^ in a comparison with the other clusters). In contrast, genes involved in antiviral responses, such as the type I IFN signaling pathway and the negative regulation of viral genome replication, are highly expressed in the other two subpopulations of A549 cells with similar or severely reduced relative abundances of virus transcripts (clusters 3 and 2, respectively, in Fig. 4a). However, some antiviral genes are differentially induced in cells with different levels of viral transcription. For example, type I and III IFNs, as well as a subset of ISGs, are highly expressed in cluster 3, and another subset of ISGs is highly expressed in cluster 2. In HBEpC, the antiviral responses, such as type I IFN signaling, are observed primarily in a cluster that has low abundances of virus transcripts (cluster 2 in Fig. 4b) and is comprised mostly of cells in the G_0_/G_1_ phase (Fig. S5b at 24 hpi), as seen for A549 cells at 12 hpi (Fig. S5a and Fig. S6).

10.1128/mBio.02880-19.6 FIG S6 Enriched biological process gene ontology (GO) terms in A549 cells at 12 hpi. The top 10 overrepresented GO terms in each cluster are denoted by bubbles. The color intensity of each bubble indicates the fold enrichment of the corresponding GO term, and the size corresponds to the ratio of queried genes in the gene set associated with a given GO term. The GO terms enriched in each cluster are color-coded by clusters. The bar chart above the bubble chart of the GO terms shows the median relative abundances of the virus transcripts across cells in each cluster. The colors of the bar are consistent with that of the GO terms. Download FIG S6, PDF file, 0.8 MB. Copyright © 2020 Wang et al. 2020 Wang et al. This content is distributed under the terms of the Creative Commons Attribution 4.0 International license.

**FIG 5:**
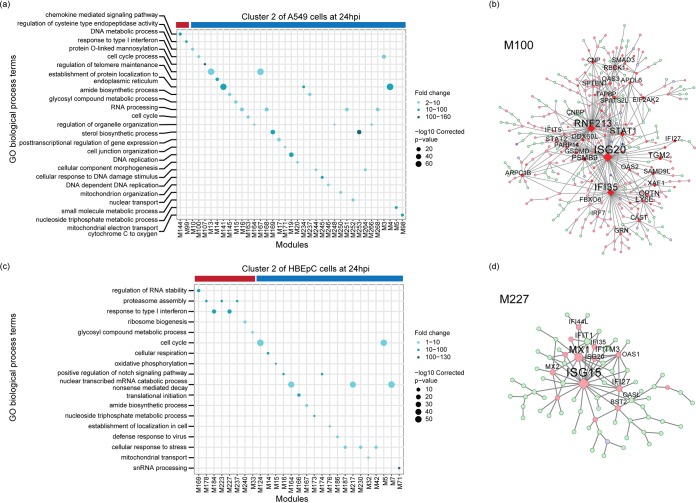
Functional MEGENA modules in clusters of A549 cells and HBEpC at 24 hpi. The left panels show enriched biological process GO terms in the modules correlated with the relative abundances of virus transcripts in cluster 2 of A549 cells (a) and cluster 2 of HBEpC (c) at 24 hpi. Each GO term is denoted by a bubble. The color intensity of each bubble indicates the fold enrichment of the corresponding GO term, and the size corresponds to the log_10_-transformed corrected *P* value for a given GO term. Red and blue color bars above the bubble charts denote positive or negative correlation of viral transcription with corresponding modules, respectively. The right panels show the MEGENA network of modules enriched with the innate immune response, including module M100 in cluster 2 of A549 cells (b) and module M227 in cluster 2 of HBEpC (d). Red and blue nodes represent significantly up- or downregulated genes compared to their expression in other cells in the population, while light-green nodes denote genes that are not significantly differentially expressed. Diamond nodes indicate key regulators. The size of the nodes indicates node strength after multiscale hub analysis within the MEGENA pipeline. Modules that have a significant correlation with the relative abundance of virus transcripts and enriched GO biological process terms are shown in the GO term bubble charts (a and c). Module member information for all viral-transcription-correlated modules can be found on GitHub (https://github.com/GhedinLab/Single-Cell-IAV-infection-in-monolayer/tree/master/AdditionalFiles/MEGENA_Tables).

To further understand how viral and host transcriptional activities are coordinated in clusters of cells that are subjected to different levels of viral stimuli, we used multiscale embedded gene coexpression network analysis (MEGENA) (48) and subsequent correlation analyses to directly characterize virus-host interactions. This was done for each cluster individually for A549 cells and HBEpC collected at 24 hpi to identify modules of coexpressed genes representing coherent functional pathways. The relevance of a module to viral infection was evaluated by correlating the first principal component of a module with the relative abundance of virus transcripts. As with the processes identified in the GO term overrepresentation approach using differentially expressed genes identified within a given cluster of cells (Fig. 4), those related to the host cell cycle and viral life cycle, including transcription, translation, RNA processing, metabolic process, and protein localization to the membrane, were enriched in the modules that were correlated with viral transcription and identified in the clusters of A549 cells with high abundances of virus transcripts (Fig. S7). Conversely, the host innate immune response (e.g., the response to type I IFN) was associated with viral-transcription-correlated modules in cells with the lowest abundance of virus transcripts (cluster 2 of A549 cells at 24 hpi in Fig. 5a). For example, module M100 negatively correlated with the relative abundance of virus transcripts (ρ = −0.262, *P* = 2.9 × 10^−5^) in cluster 2 of A549 cells, which has genes, including *ISG20*, *RNF213*, *IFI35*, *STAT1*, *RBCK1*, *IFI27*, *OAS2*, *OAS3*, *XAF1*, *STAT2*, and *IFIT5*, that are involved in the innate antiviral response and that were significantly highly expressed compared to their expression in other cells of the same population (Fig. 5b). In HBEpC, viral-transcription-correlated modules, such as M227, which were enriched for type I IFN signaling genes (e.g., *ISG15*, *MX1*, *MX2*, *IFI35*, *IFI27*, *IFIT1*, *IFITM3*, *OAS1*, *OASL*, and *ISG20*) and positively correlated with the relative abundance of virus transcripts (ρ = 0.103, *P* = 0.021) (Fig. 5c and d), were detected in one cluster of cells with a low abundance of virus transcripts (cluster 2 of HBEpC at 24 hpi in Fig. 5c). Modules correlated with viral transcription in the other two clusters of HBEpC with low abundances of virus transcripts (clusters 0 and 1 in Fig. S8) were enriched for genes associated with the host cell cycle, consistent with observations of A549 cells at 12 hpi (Fig. S6), while the viral-transcription-correlated modules in HBEpC with a high abundance of virus transcripts (cluster 3 in Fig. S8) were enriched for genes involved in the endoplasmic reticulum (ER)-associated ubiquitin-dependent protein catabolic process, the small-molecule metabolic process, and vesicle-mediated transport, to name a few. Overall, by identifying modules of genes that have a direct correlation with virus transcript abundance, the gene coexpression network analysis further demonstrates a complex association between virus transcript abundance and host responses, consistent with the pattern revealed by GO term analysis of highly expressed genes in individual clusters, and provides further evidence for the complexity of virus-host interactions.

10.1128/mBio.02880-19.7 FIG S7 Functional MEGENA modules in clusters 0 and 1 of A549 at 24 hpi. Each panel shows enriched biological process GO terms in the modules correlated with the relative abundances of virus transcripts in corresponding clusters. Each GO term is denoted by a bubble. The color intensity of each bubble indicates the fold enrichment of the corresponding GO term, and the size corresponds to the log_10_-transformed corrected *P* value for a given GO term. Red and blue color bars above the bubble charts denote positive and negative correlations of viral transcription with the corresponding modules, respectively. Modules that have a significant correlation with the relative abundances of virus transcripts and enriched GO biological process terms are shown in the GO term bubble charts. Module member information for all viral transcription-correlated modules can be found on GitHub (https://github.com/GhedinLab/Single-Cell-IAV-infection-in-monolayer/tree/master/AdditionalFiles/MEGENA_Tables). Download FIG S7, PDF file, 0.8 MB. Copyright © 2020 Wang et al. 2020 Wang et al. This content is distributed under the terms of the Creative Commons Attribution 4.0 International license.

10.1128/mBio.02880-19.8 FIG S8 Functional MEGENA modules in clusters 0, 1, and 3 of HBEpC at 24 hpi. Each bubble chart shows enriched biological process GO terms in the modules correlated with the relative abundances of virus transcripts in corresponding clusters. Each GO term is denoted by a bubble. The color intensity of each bubble indicates the fold enrichment of the corresponding GO term, and the size corresponds to the log_10_-transformed corrected *P* value for a given GO term. Red and blue color-bars above the bubble charts denote positive or negative correlation of viral transcription with corresponding modules, respectively. Modules that have a significant correlation with the relative abundance of virus transcripts and enriched GO biological process terms are shown in the GO term bubble charts. Module member information for all of viral transcription-correlated modules can be found on GitHub (https://github.com/GhedinLab/Single-Cell-IAV-infection-in-monolayer/tree/master/AdditionalFiles/MEGENA_Tables). Download FIG S8, PDF file, 0.8 MB. Copyright © 2020 Wang et al. 2020 Wang et al. This content is distributed under the terms of the Creative Commons Attribution 4.0 International license.

### Differential expression of host genes linked to defective viral genomes.

**FIG 6:**
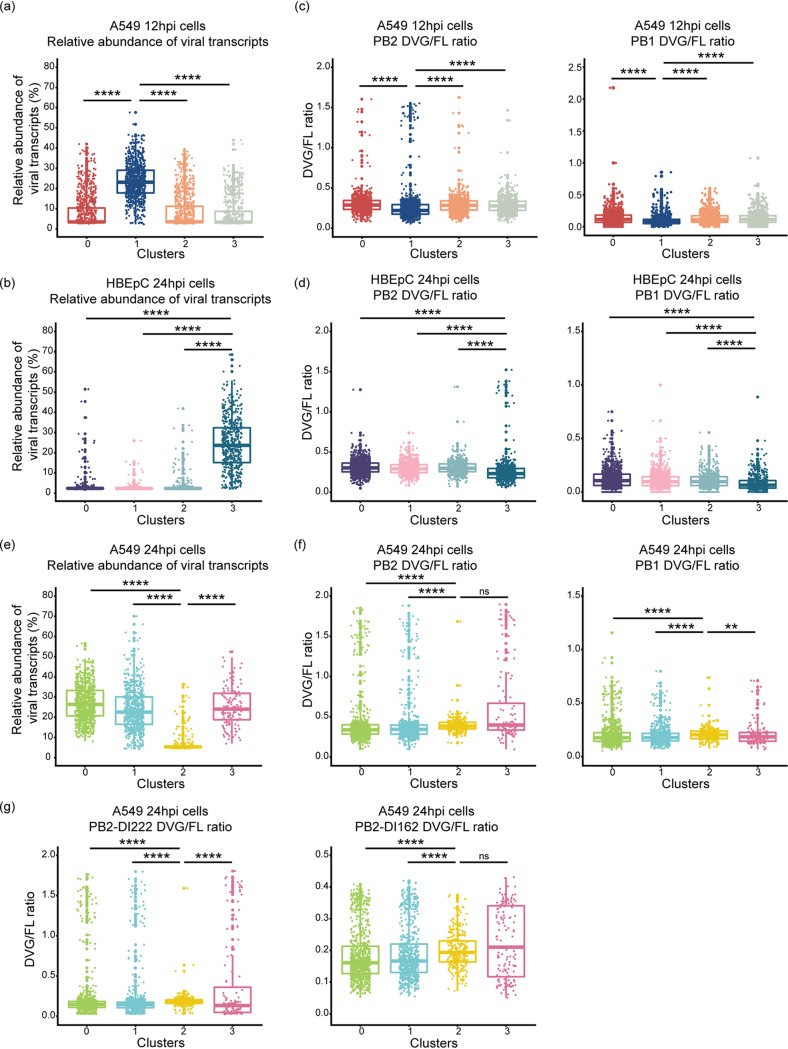
Distribution of the relative abundances of virus transcripts and DVG/FL ratios for DVG PB2 and PB1 transcripts in each cluster of cells. (a, b, and e) Box plots of the relative abundances of virus transcripts in each cluster of A549 cells at 12 hpi (a) and at 24 hpi (e) and of HBEpC at 24 hpi (b). (c, d, and f) DVG/FL ratios for DVG PB2 and PB1 transcripts calculated as the ratio of gap-spanning reads to non-gap-spanning reads aligned to a 3′ coverage peak region derived from the corresponding viral segments in each cluster. (g) DVG/FL ratios for PB2-DVG transcripts carrying deletion sites similar to those in PR8-DI222 and PR8-DI162 viruses in each cluster of A549 cells at 24 hpi. All box plots show the first and third quantiles as the lower and upper hinges, the median in the center, and a 1.5 interquartile range (IQR) from the first and third quantiles as whiskers. Asterisks over the boxes denote the statistical significance levels of pairwise comparisons determined by the one-tailed Wilcoxon rank sum test: *, *P* ≤ 0.05; **, *P* ≤ 0.01; ***, *P* ≤ 0.001; and ****, *P* ≤ 0.0001. ns, not significant.

Since DVGs are known to play an important role in IFN induction and inhibition of viral replication (reviewed in references [Bibr B28][Bibr B29][Bibr B30] and 36), we determined whether the accumulation of DVGs was associated with attenuated viral transcription and induction of innate immune antiviral response genes. We observed a subtle but consistent inverse relationship between the relative abundance of virus transcripts and the PB2 and PB1 DVG/FL ratios within each cluster of A549 cells at 12 hpi and that of HBEpC at 24 hpi (Fig. 6a to d). The cluster of cells with the highest abundance of virus transcripts (cluster 1 of A549 cells at 12 hpi in Fig. 6a and cluster 3 of HBEpC at 24 hpi in Fig. 6b) had the lowest PB2 and PB1 DVG/FL ratios (Fig. 6c and d) (*P* was <2.2 × 10^−16^ in a comparison of PB2 and PB1 DVG/FL ratios in cluster 1 of A549 cells or cluster 3 of HBEpC against the other clusters). However, in A549 cells at 24 hpi, higher PB2 DVG/FL ratios were observed in the cluster of cells with the lowest abundance of virus transcripts (cluster 2 in Fig. 6e and f) (*P* was <2.2 × 10^−16^ in a comparison with clusters 0 and 1 in Fig. 6f) and in a cluster of cells with a relatively high abundance of virus transcripts (cluster 3 in Fig. 6e and f) (*P* was 4.036 × 10^−11^ in a comparison of clusters 0 and 1 in Fig. 6f). The highest level of PB1 DVG/FL ratios was observed only in cluster 2 (*P* was 1.943 × 10^−9^ in a comparison of cluster 2 with the other clusters). Moreover, innate immune response genes were highly expressed in cells with either an elevated DVG/FL ratio for both DVG PB2 and PB1 transcripts and a low abundance of virus transcripts (cluster 2 of A549 cells at 24 hpi in Fig. 6e and f) or an elevated DVG/FL ratio for PB2 transcripts and a relatively high abundance of virus transcripts (cluster 3 in Fig. 6e and f). It suggests that both DVGs and full-length genomes may play a role in stimulating the innate immune response and that DVG immunostimulation may also be independent of viral abundance. Notably, the abundances of two dominant and ubiquitous PB2-DVG transcripts in the total virus transcript pool vary between cells and across clusters. The PB2-DVG transcripts carrying the same deletion sites as in PR8-DI222 were highly abundant in the cluster of cells with the lowest abundance of virus transcripts (cluster 2 of A549 cells at 24 hpi in Fig. 6g) (*P* was <2.2 × 10^−16^ in a comparison with the other clusters), while the other PB2-DVG transcripts corresponding to PR8-DI162 were highly abundant in both cluster 2 and cluster 3 (Fig. 6g) (the *P* values were 2.067 × 10^−10^ and 0.001241, respectively, in comparisons with clusters 0 and 1), suggesting a potential difference in the levels of induction of innate immune response genes by different DVG species. We did not detect a difference in the relative abundances of defective PA transcripts among cells with different levels of viral transcription (Fig. S9).

10.1128/mBio.02880-19.9 FIG S9 Distribution of the DVG/FL ratios for the DVG PA transcripts in each cluster of A549 cells at 12 hpi and 24 hpi and in HBEpC at 24 hpi. All box plots show the first and third quantiles as the lower and upper hinges, the median in the center, and a 1.5 interquartile range (IQR) from the first and third quantiles as the whiskers. The significance levels of pairwise comparisons determined by one-tailed Wilcoxon rank sum test were denoted by the asterisks as follows: *, *P* ≤ 0.05; **, *P* ≤ 0.01; ***, *P* ≤ 0.001; and ****, *P* ≤ 0.0001. ns, not significant. Download FIG S9, PDF file, 1.8 MB. Copyright © 2020 Wang et al. 2020 Wang et al. This content is distributed under the terms of the Creative Commons Attribution 4.0 International license.

Given the enrichment of PB2-DVGs and the overexpression of ISGs in cluster 2 of A549 cells at 24 hpi compared to their representation in the rest of the cells in the same population, we experimentally validated the immunostimulatory impact of PB2-DVGs on the host cells. We infected A549 cells with each of the 3 PR8-DIs (PR8-DI162, -DI222, or -DI244) or with WT virus. We quantified the expression levels of the influenza virus M gene, of ISGs that were shown to be significantly highly expressed in cluster 2 of A549 cells, including *IFI35*, *IFI27*, and *MX1*, and of the type I (IFN-β) and III (IFN-λ) IFNs that were shown to be significantly highly expressed in cluster 3 of A549 cells (Fig. S10a and b). Due to their limited ability to replicate without a helper virus, DIs cultured on their own exhibit a significantly lower level of viral transcription than WT virus (Fig. S10c). Despite the lower replication of DIs, there was a higher induction of ISGs in DI-infected cells than in cells infected with WT virus, while there were comparable or marginally higher inductions of IFN-β and IFN-λ in the WT-infected cells (Fig. S10c). Together, these data demonstrate that at the late stage of infection, PB2-DVGs have a strong immunostimulatory effect on the host cells manifested by a significant induction of ISGs, even at very low levels of viral transcription and independently of type I and III IFN expression levels.

10.1128/mBio.02880-19.10 FIG S10 Expression of ISGs and IFNs in DI-enriched A549 cells at 24 hpi. (a and b) Expression levels of significantly overexpressed genes in cluster 2, which are associated with GO terms related to the type I IFN signaling pathway and have fold changes on a log scale of ≥1 (a), and of type I and III IFNs in each cluster of A549 cells at 24 hpi (b). The dot represents the normalized expression level of a gene in each cell. The violin color is determined by the cluster identity. (c) Validation of overexpression of selected ISGs and IFNs and the M gene of IAV (i.e., FLU) by qPCR. Subconfluent A549 cells were either mock infected or infected with one type of PR8-DI (PR8-DI162, -DI222, or -DI244) or PR8-WT virus at an MOI of 10. Total RNA was extracted from cells collected at 24 hpi. The levels of ISGs, IFNs, and the IAV M gene mRNA were determined by qPCR and normalized to the level of β-actin (ACTB). The error bar representing the standard deviation of the mean was obtained from three biological replicates. Statistical tests were done using the two-tailed Student *t*-test in R to compare the differences in fold change between PR8-WT and PR8-DI infection. The significance levels are denoted by the asterisks as follows: *, *P* ≤ 0.05; **, *P* ≤ 0.01; ***, *P* ≤ 0.001; and ****, *P* ≤ 0.0001. ns, not significant. Download FIG S10, PDF file, 2.9 MB. Copyright © 2020 Wang et al. 2020 Wang et al. This content is distributed under the terms of the Creative Commons Attribution 4.0 International license.

## DISCUSSION

The goal of this study was to quantitatively and qualitatively characterize viral and host factors that contribute to cell-to-cell transcriptional variation observed during IAV infection. We identified DVGs as potentially important factors in the temporal variation of the host cell transcriptome. The substantial cell-to-cell variation in IAV replication and host response that we detected highlights how single-cell RNA-seq experiments can provide novel insight into the complexity of virus-host (and DI-host) interactions in influenza virus infection.

The inherent stochasticity of molecular processes, especially during the initial stages of infection, can contribute to the cell-to-cell variation in viral replication (1). Evidence from studies examining low-MOI IAV infections with different virus strains and cell lines suggests that this intrinsic stochasticity can impact viral replication and result in the loss of viral genome segments, transcripts, or proteins (1, 11, 12, 16). Similarly, at different time points of the high-MOI infection, we observed cell-to-cell variation in viral transcription, including segment-specific transcription. Although the mechanisms leading to this observation are unclear based on our data, it may be explained by the following possibilities as discussed in reference 16, including (i) the absence of one or more viral genome segment in infecting virions, (ii) a deficiency in intracellular trafficking of incoming viral ribonucleoprotein complexes (vRNPs), (iii) the random degradation of vRNAs prior to transcription, or (iv) mutations resulting in the decreased stability of viral mRNAs.

Another source of variability in viral replication stems from the emergence and accumulation of various vRNA species synthesized by the error-prone viral polymerase (reviewed in references 49 and 50). Indeed, viral genetic diversity, including amino acid mutations in the NS1 and PB1 proteins, the absence of the NS gene, and an internal deletion in the PB1 gene, contributes to the cell-to-cell variation in the innate immune response, as shown in a recent report on a low-MOI infection (13). Although the 3′ sequencing strategy that we used in our study limits the identification of mutations in the full-length segments, our experimental setup provides a unique opportunity to evaluate the diversity of DVGs by characterizing DVG transcripts, since a high-MOI infection promotes the accumulation of DVGs. Late stages of infection also emphasize the impact of accumulated DVGs on the host response. We identified a diverse pool of DVG transcripts derived from the three polymerase segments, including several dominant species. Notably, the fact that all the dominant DVG PB2 and PB1 species identified in the infected cells were also present in the virus stock and that the dominant PB1 DVG species were detected in an increasing proportion of cells over the course of the infection suggest potential cellular transmission of DIs carrying these DVGs. However, we cannot exclude the possibility that the regions where the hot spots were located are most prone to polymerase skipping and that DVG enrichment over the course of the infection resulted in their abundance above a detection threshold in an increasing number of cells.

The accumulation of DVGs can have consequences on viral replication by directly competing with the full-length genomes and modulating host innate immune responses (reviewed in references 27 to 30, 49, and [Bibr B51][Bibr B52][Bibr B53]). The immune-modulatory effect of DVGs has been attributed to efficient activation of the IFN induction cascade and antiviral immunity, a mechanism especially well known for the copy-back DVGs found in many negative-sense RNA viruses, such as SeV (reviewed in references 28 to 30 and 36). Consistently with previous reports, we detected enrichment of highly expressed genes involved in the IFN response in one subpopulation of cells with significantly attenuated viral transcription. In this cluster of cells, we also observed that DVG transcripts accumulated to a high level. The observation was further validated in our DI or WT infection assay, in which we detected a significantly higher expression of some ISGs in DI-infected cells independently of the level of viral transcription and of IFNs at the late stage of the infection. The observed strong innate immune response in DI-infected cells may be explained by reduced expression of the NS1 protein, a major IFN antagonist, compared to that in WT-infected cells. However, the observation of DI-mediated antiviral protection against lethal homologous WT virus infection in type I IFN receptor knockout mice suggests the involvement of innate immune factors other than type I IFN (54). Alternatively, the IFN mRNA dose-independent ISG expression in DI-infected cells may suggest a potential novel mechanism of DVG immunostimulation that is independent of IFN induction. Expression of a subset of ISGs can be induced in the absence of IFN signaling (55, 56). It is likely to be at least partially attributed to similar and overlapping consensus DNA-binding motifs for the interferon regulatory factor (IRF) family, most notably IRF3 and IRF7, which are key regulators of type I IFN production, and for IFN-stimulated gene factor 3 (ISGF3), which promotes the expression of many ISGs in response to IFN signaling (57–60). For example, an IRF3 chromatin immunoprecipitation sequencing (ChIP-seq) experiment with adenovirus-infected human primary bronchial/tracheal epithelial cells demonstrated IRF3 binding to the promoter region of induced ISGs, including MX1 and ISG15, which have the IFN-stimulated response element (ISRE) bound by IRFs and ISGF3 in the promoter regions (61). Further investigation of ISG induction in the absence of IFN signaling during DI infection will determine whether an IFN-independent mechanism of DI immunostimulation does indeed exist. In addition, given the fact that some ISGs may be highly expressed in cells with a high level of viral transcription or a high abundance of DVGs in the virus pool suggests that viral factors other than the accumulation of DVGs may also play an important role in triggering the innate immune response. Nevertheless, unlike SeV copy-back DVGs, which carry complementary termini generated by copying back the authentic 5′ terminus to the 3′ terminus (62, 63), IAV “deletion” DVGs have termini identical to those of the full-length genomes. In the SeV copy-back DVGs, a stretch of double-stranded RNA (dsRNA) adjacent to a 5′ triphosphate serves as an effective RIG-I ligand and strongly activates IFN-β (35, 36, 63, 64), and an RNA motif presented in the DVGs plays an important role in stimulating a strong antiviral response (65, 66). In influenza virus infection, short viral RNA templates (shorter than 149 nt) retaining the conserved 5′ and 3′ termini, called mini vRNAs (mvRNAs) (67), can be transcribed and replicated in the absence of nucleoprotein (NP) (68) and induce strong IFN-β expression by binding to, and activating, RIG-I (67). However, the mechanism of a more effective immunostimulation by IAV DVGs (which are larger than mvRNAs) than by the full-length genomes remains elusive (reviewed in reference 36). In addition, the interfering and immune-modulatory abilities of influenza virus DVGs may be attributed to DVG-encoded proteins that directly interact with mitochondrial antiviral signaling (MAVS) and act independently of RIG-I in IFN induction, as previously reported (69).

Cell-to-cell variation during viral infection has been reported in studies of different viruses using different single-cell techniques (1, 2, 4–10, 12–15, 70–74). In the past few years, dissecting heterogeneity of viral replication and understanding complex virus-host interactions have been greatly facilitated by the application of fast-evolving single-cell RNA-seq approaches (reviewed in reference 75). Our study, along with several other *in vitro* (12, 13, 15) and *in vivo* (14) studies of single-cell transcriptome dynamics during influenza virus infection, reveals substantial heterogeneity of viral gene expression and cellular responses, especially the innate antiviral response. However, unlike other *in vitro* studies using single-cell RNA-seq (12, 13, 15) that focused either on a low-MOI infection scenario, which limits the chance of coinfection, or on the early-to-middle stages of a high-MOI infection, which emphasizes the early host response, our study of high-MOI infection over multiple infection stages allows us to characterize and determine the abundances of diverse DVG species that accumulated in individual cells and to comprehensively capture variations in host responses in these infected cells. By distinguishing different DVG species at the sequence level, it also allowed us to associate the enrichment of certain DVG species with the attenuation of viral transcription and the induction of innate immune responses at a single-cell resolution. For example, the accumulation of PB2-DVGs in one subpopulation of cells was associated with significantly attenuated viral transcription and a strong host innate immune response manifested by high expression of some ISGs. Although this appeared to be associated primarily with one dominant PB2-DVG species (PR8-DI222), we cannot rule out the effect of other non-dominant defective PB2-DVGs, DVGs derived from other segments, or certain mutations in stimulating host responses. Diverse DVG species can play different roles in shaping the virus-host interaction. Further investigation is necessary to elucidate the dynamics and impact of diverse DVG species during influenza virus pathogenesis. Our characterization of DVGs and corresponding host responses at the single-cell level provides a different view into complex virus-host interactions and demonstrates the intricate effects of various viral factors, including (but probably not limited to) the accumulation of DVGs, in shaping infection outcome.

## MATERIALS AND METHODS

### Cells and virus.

Human lung adenocarcinoma epithelial A549 cells (ATCC, VA, USA) were maintained in Kaighn’s modified Ham’s F-12 medium (F-12K) supplemented with 10% fetal bovine serum (FBS). Primary human bronchial epithelial cells (HBEpC) (PromoCell, Heidelberg, Germany) were maintained in PromoCell airway epithelial cell growth medium with SupplementMix (PromoCell). Madin-Darby canine kidney (MDCK) cells were maintained in minimum essential medium (MEM) supplemented with 5% FBS. Human embryonic kidney epithelial 293T cells (ATCC, VA, USA) were maintained in Dulbecco’s modified Eagle medium (DMEM) supplemented with 10% FBS. PB2 protein-expressing modified MDCK (AX4/PB2) (76) cells were maintained in MEM supplemented with 5% newborn calf serum (NBCS), 2 μg/ml puromycin, and 1 μg/ml blasticidin. 293T and AX4/PB2 cells were cocultured with DMEM supplemented with 10% FBS. Viral strain A/Puerto Rico/8/34 (H1N1) was plaque purified and propagated in MDCK cells. Viral titers were determined by plaque assay in MDCK cells and sequences confirmed by Illumina MiSeq sequencing.

### Infection assays.

Subconfluent monolayers of A549 cells in T-25 flasks were washed with A549 infection medium (F-12K supplemented with 0.15% bovine serum albumin [BSA] fraction V and 1% antibiotic-antimycotic) and infected with influenza virus at a multiplicity of infection (MOI) of 5 in 0.5 ml precooled A549 infection medium. The flasks were incubated at 4°C for 30 min with agitation every 5 to 10 min, followed by the addition of 4.5 ml prewarmed A549 infection medium, and transferred to a 37°C, 5% CO_2_ incubator for further incubation. HBEpC were infected similarly except that the HBEpC infection medium was PromoCell airway epithelial cell growth medium. The inoculum was back-titrated to confirm that the desired MOI was used. The virus-infected cells were collected at 6, 12, and 24 hpi, and the mock-infected cells were collected at 12 hpi. There was no substantial cell death observed at any time point. Cells were extensively washed before their resuspension in PBS containing 0.04% BSA. A proportion of cells from one of the two duplicate flasks per time point were subject to 10× Genomics Chromium single-cell library preparation. The remaining cells in the two flasks were used for conventional bulk RNA-seq library preparation. Notably, due to a failure in the single-cell library preparation for the PR8-infected HBEpC collected at 12 hpi, we replaced this sample by repeating the same infection assay with HBEpC.

### Sample preparation, library construction, and sequencing.

A 10× Genomics single-cell library preparation with Chromium 3′ v2 chemistry was performed by following the manufacturer’s protocol. A range of 3,900 to 7,500 cells were used as the input in each single-cell preparation, with a median of ∼3,353 cells (range, 2,075 to 5,254 cells) obtained following sequencing, as described below. Sequencing was performed on the HiSeq 2500 in HighOutput mode (v2) with one library per lane according to the manufacturer’s recommended sequencing configuration (i.e., for paired-end read 1, 27 bp, for read 2, 99 bp, and for the i7 index, 8 bp). An average of 59,200 reads per cell was obtained. For bulk mRNA sequencing, total RNA from two replicate flasks per time point was extracted using an RNeasy minikit (Qiagen, Hilden, Germany) with on-column DNA digestion using RNase-free DNase (Qiagen). The conventional bulk RNA-seq library was prepared using an NEBNext Ultra II RNA library prep kit for Illumina following poly(A) mRNA enrichment. Libraries were multiplexed and sequenced on an Illumina NextSeq 500 in HighOutput 2× 75-bp mode (v3). Viral genomic RNA (vRNA) of the virus stocks used in the infection assay was amplified using multisegment reverse transcription-PCR (M-RTPCR) (77). The M-RTPCR product was subject to Illumina library preparation with a Nextera DNA library prep kit and sequenced on the HiSeq 2500 in rapid 2× 150-bp mode (v2).

### Upstream computational analyses of single-cell RNA-seq data.

The Cell Ranger (v2.1.0) single-cell software suite (10× Genomics) was applied to the single-cell RNA-seq data to perform the alignment with the concatenated human (hg19) and influenza A virus (A/Puerto Rico/8/34) references with STAR (v2.5.3a) (78) and gene counting with the processed unique molecular identifiers (UMIs) for each cell. The UMI counts for the viral reads with the CIGAR string containing a gap (i.e., MNM) and different UMI sequences for comparing to the ungapped reads were added into the expression matrix later, since they were not considered during gene counting. Given the fact that we performed 3′-end sequencing, transcripts derived from alternative splicing of M and NS segments, encoding M1/M2 and NS1/NEP, respectively, could not be distinguished in our analyses, as those transcribed from the same genes have identical 3′ ends.

To identify and exclude low-quality cells in the single-cell data from the downstream analyses, the following steps were taken for each sample and were employed to filter out cells that failed to meet these criteria: we (i) removed cells with fewer than 2,000 detected genes, (ii) removed cells with an alignment rate of less than the mean minus 3 standard deviations, (iii) removed cells for which the number of reads after log_10_ transformation was not within 3 standard deviations below or above the mean, (iv) removed cells for which the number of UMIs after log_10_ transformation was not within 3 standard deviations below or above the mean, (v) removed cells for which the percentage of reads aligned with mitochondrial genes was not within 3 standard deviations below or above the mean, and (vi) removed mock-infected cells with 1 viral read detected. Although we filtered cells based on the size of the total mRNA pool (i.e., total reads) as one of the criteria described above, we also checked the distribution of the size of the host mRNA pool (i.e., host reads) for individual cells. As reported in recent virus-infected single-cell studies (12, 14, 15), while virus-induced host shutoff of gene expression (79) is likely to be mediated mainly by host mRNA degradation, it remains unclear what effect viral transcription has on the size of the total mRNA pools. In our study, we observed that virtually all the cells with small or large host mRNA pools for which the number of host reads after log_10_ transformation was not within 3 standard deviations below or above the mean were already being excluded when the 6 criteria described above were applied; thus, we decided to filter cells based on these. Approximately 120 to 250 cells were eliminated from each sample, with a median of 164 cells per sample. We further removed genes that were detected in fewer than three cells. After initial cell and gene quality control, the majority of samples had approximately 3,000 to 3,500 cells left for downstream analysis. The expression levels of host genes were normalized based on the size of the host mRNA pool.

### Cell clustering with Seurat.

An unsupervised cell clustering to identify subtle changes in the population structure after infection was performed on each time point datum separately by following the procedures of the Seurat package (v2.1.0) (80) using the normalized, scaled, and centered host gene expression matrix, without consideration of the relative abundances of virus transcripts within individual cells. Briefly, the highly variable genes with average levels of expression of <4 and dispersion of >1 were used as input for the principal-component analysis (PCA). The statistically significant and biological meaningful PCs determined by the built-in jackstraw and elbow analyses and manual exploration were retained for visualization by *t*-distributed stochastic neighbor (t-SNE) and subsequent clustering by a shared nearest neighbor (SNN) graph-based approach. The legitimacy of the initially identified clusters was validated using the ValidateClusters function in Seurat, which built a support vector machine (SVM) classifier with significant PCs and then applied the accuracy cutoff of 0.9 and the minimal connectivity threshold of 0.001. To identify markers that are differentially expressed among clusters, the FindMarkers function in Seurat was used with the different test options, including bimod (81), poisson, negbinom, and MAST (v1.4.1) (82). Only the genes that showed at least a 0.25-fold difference on the log scale between two groups and were expressed in at least 25% of the cells in either group were tested for differential expression. Significantly differentially expressed genes for each cluster (i.e., the markers) were identified by applying the adjusted *P* value cutoff of 0.05. Markers identified by all four methods were retained. GO term overrepresentation analysis of the upregulated markers was performed with the online service DAVID (83, 84) by applying the Benjamini *P* value cutoff of 0.05. To determine the cell cycle stage associated with individual cells, cell cycle scoring and assignment were performed with Seurat using the CellCycleScoring function based on the expression of canonical markers (85).

### Computational analyses of bulk RNA-seq and virus stock sequencing data.

The raw bulk RNA-seq and virus stock sequencing data were first trimmed with Trimmomatic (v0.36) (86) to remove the adaptors and trim off low-quality bases. Reads with a minimal length of 36 bases in the trimmed bulk RNA-seq data set were aligned to the concatenated human (hg19) and influenza A/Puerto Rico/8/34 (H1N1) references with STAR (v2.5.3a) (78) with the default parameters and counted with featureCounts (87) in the Subread package (v1.5.1) (88), while reads in the virus stock sequencing data set were aligned to the influenza A/Puerto Rico/8/34 (H1N1) reference with STAR (v2.5.3a) (78). Differential-expression analysis was performed with DESeq2 (v1.18.1) (89) and edgeR (v3.20.5) (90, 91) using the bulk RNA-seq and the merged single-cell RNA-seq data. Host genes with the adjusted *P* value of <0.05 were identified as significantly differentially expressed at each time point.

### Deletion junction identification, filtering, and quantification.

Reads that aligned to both ends of a viral segment, with each aligned portion comprised of at least 10 nucleotides in length, were collected. Following UMI deduplication, reads with junction coordinates within a 10-nucleotide window were grouped together. We excluded from downstream analyses reads with junction coordinates that occurred fewer than 10 times in each sample. To compare quantitatively gap-spanning reads for each segment across cells and samples, the number of gap-spanning reads was normalized to the total number of non-gap-spanning reads aligned to a 100-nt region, with the coverage peak centered at the 3′ end. For the polymerase segments, gap-spanning reads were also filtered by the size of deletions (i.e., ≥1,000 nucleotides). The 1,000-nucleotide threshold for the polymerase segments was chosen based on the distribution of the size of the deletions (data are available on GitHub, https://github.com/GhedinLab/Single-Cell-IAV-infection-in-monolayer/blob/master/AdditionalFiles/AdditionalFile5_polymerase-DVG-deletion-size-distribution.pdf), as the majority of the deletions are larger than 1,000 nucleotides. Because cells with low infection (especially those harvested at the early stage of infection or inoculated at a low MOI) typically have poor coverage for the viral segments of interest, the DVG/FL ratio in those cells calculated as described above is typically inflated and may even fail to be calculated because all the viral reads corresponding to a given segment span gaps or there are no viral reads. To mitigate this effect, we overwrote those values with 0 in the data sets, including the data set collected from HBEpC at 6 hpi.

### Generation of PR8-derived DI162, DI222, DI244, and PB2del virus.

To generate the PR8 PB2-DI162, -DI222, and -DI244 reverse-genetics plasmids, gBlocks gene fragments (Integrated DNA Technologies, CA, USA) were ordered using the corresponding defective PB2 genomic sequences identified in this study (PR8-DI162 and PR8-DI222) or previously reported (DI244) (31). To generate the PR8 PB2del plasmid, a point mutation at nucleotide position 265 was introduced into the PR8 PB2 genomic sequence using the mutagenesis primer PR8-PB2-stopdel-F (5′-TTCATTTACTCCATTAAGTTTGTCCTTGCTC-3′). The reverse-genetics plasmids were cloned into the pBZ61A18 reverse-genetics vector as previously described (92). To rescue the PR8 DIs, each of the sequence-confirmed PB2-DI or PB2del plasmids was cotransfected into cocultured 293T-AX4/PB2 cells with the 7 reverse-genetics plasmids for PR8 PB1, PA, HA, NP, NA, M, and NS and a PB2 protein expression plasmid using Lipofectamine 3000 transfection reagent (Invitrogen, CA, USA). The supernatant was collected on day 2 postinfection and passaged twice in the AX4/PB2 cell line, which expresses the PB2 protein in *trans* (76). Viral titers were determined by a 50% tissue culture infective dose (TCID_50_) assay in AX4/PB2 cells.

### Interference test for PR8-DI162 and PR8-DI222 viruses and validation of their immunostimulatory effects.

To test the interfering ability of PR8-DI162 and PR8-DI222 viruses that carry the deletions identified in two dominant PB2-DVG species from the single-cell data set, their inhibitory effects on wild-type PR8 virus replication were quantified in cultured cells at different DI/WT ratios. Subconfluent monolayers of A549 cells in 12-well plates were washed with infection medium (MEM supplemented with 0.15% BSA fraction V, 1% antibiotic-antimycotic, and 1 μg/ml tosylsulfonyl phenylalanyl chloromethyl ketone [TPCK]-treated trypsin), and each well was coinfected with one type of defective virus at an MOI of 5 and the PR8 virus either at an MOI of 0.005 for a DI/WT ratio of 1,000:1 or at an MOI of 5 for a DI/WT ratio of 1:1 in 1 ml infection medium. For a DI/WT coinfection ratio of 1,000:1, plates were transferred to a 37°C, 5% CO_2_ incubator and supernatants were collected at 2 h postinfection and 1, 2, and 3 days postinfection. For a DI/WT coinfection ratio of 1:1, infection was similar to that used for single-cell RNA-seq, including incubation at 4°C for 30 min with agitation every 5 to 10 min after infection followed by addition of prewarmed A549 infection medium, and cells were transferred to a 37°C, 5% CO_2_ incubator for further incubation. Supernatants were collected at 2, 6, 12, and 24 h postinfection and titrated as described above. The wild-type PR8 virus yield from collected supernatants was determined by a TCID_50_ assay using MDCK cells, which do not support replication of DIs due to the lack of functional PB2 proteins.

To determine if the DIs could stimulate a higher expression of some ISGs identified in the network analysis, subconfluent monolayers of A549 cells in 12-well plates were infected with each DI (PR8-DI162, -DI222, or -DI244) or WT virus at an MOI of 10 in triplicate. Total RNA from cells collected at 24 hpi was extracted using an RNeasy minikit (Qiagen, Hilden, Germany). One hundred nanograms of RNA was subsequently used as the template for reverse transcription using the SuperScript IV system (Invitrogen, CA, USA) with an oligo(dT)_20_ primer. Quantitative PCR (qPCR) was performed using PowerUp SYBR green (Applied Biosystems, MA, USA) in triplicate with the following primers: for IFI35, Hs.PT.58.38490206 (IDT, IA, USA); for IFI27, IFI27fw (5′-GCCTCTGGCTCTGCCGTAGTT-3′) and IFI27rev (5′-ATGGAGGACGAGGCGATTCC-3′) (93); for MX1, MX1fw (5′-CTTTCCAGTCCAGCTCGGCA-3′) and MX1rev (5′-AGCTGCTGGCCGTACGTCTG-3′) (93); for IFN-β, IFNBfw (5′-TCTGGCACAACAGGTAGTAGGC-3′) and IFNBrev (5′-GAGAAGCACAACAGGAGAGCAA-3′) (94); for IFN-λ, IFNLfw (5′-GCCCCCAAAAAGGAGTCCG-3′) and IFNLrev (5′-AGGTTCCCATCGGCCACATA-3′) (94); for β-actin (ACTB), ACTBfw (5′-GAACGGTGAAGGTGACAG-3′) and ACTBrev (5′-TTTAGGATGGCAAGGGACT-3′) (95); and for FLU, mCDC-FluA-F-PR8 (5′-GACCAATCCTGTCACCTCTGA-3′) and mCDC-FluA-R (5′-AGGGCATTTTGGACAAAGCGTCTA-3′). For all the targets, qPCR parameters, according to the manufacturer’s recommendation, were 95°C for 10 min and then 45 cycles of 95°C for 15 s, 57°C for 15 s, and 60°C for 60 s. The fold change of target gene expression was calculated using the $2^{-\Delta \Delta C_{T}}$ method (where *C_T_* is the threshold cycle), normalized to the value for ACTB.

### Gene coexpression network analysis.

Multiscale embedded-gene coexpression network analysis (MEGENA) (48) was performed to identify host modules of highly coexpressed genes in influenza virus infection. The MEGENA workflow comprises 4 major steps: (i) fast planar-filtered-network construction (FPFNC), (ii) multiscale clustering analysis (MCA), (iii) multiscale hub analysis (MHA), (iv) and cluster-trait association analysis (CTA). A cutoff of 0.05 after perturbation-based false-discovery rate (FDR) calculation was used. The differentially expressed genes are identified between virus-infected cells of a particular cluster and the rest of the cells in the same population, determined by a *t* test. Only the genes that showed at least a 0.25-fold difference on the log scale between two groups and were expressed in at least 25% of the cells in either group were tested for differential expression. Significantly differentially expressed genes were identified by applying the adjusted *P* value cutoff of 0.05.

### Identification of enriched GO terms, key regulators in the host module, and relative abundances of virus transcripts associated with host modules.

To functionally annotate gene signatures and gene modules identified in this study, enrichment analysis of the established gene ontology (GO) categories was performed. The hub genes in each subnetwork were identified using the adopted Fisher’s inverse chi-square approach in MEGENA; Bonferroni-corrected *P* values smaller than 0.05 were set as the threshold to identify significant hubs.

The relative abundances of virus transcripts and the DVGs associated with the host modules were identified using Spearman correlation between the first principal component of the gene expression in the corresponding module and the relative abundances of virus transcripts. Significantly associated traits were identified using the Benjamini-Hochberg FDR-corrected *P* value of 0.05 as the cutoff.

### Statistical analysis.

The statistical significance of the changes in the relative abundances of viral and DVG transcripts between 3 or more groups of cells was first determined by the one- or two-tailed Kruskal-Wallis rank sum test, followed by the one-tailed Wilcoxon rank sum test to calculate the pairwise comparisons. The statistical significance of expression fold changes in the qPCR validation assay was determined using the two-tailed Student *t* test. A *P* value of ≤0.05 was considered statistically significant.

### Data availability.

The code used to generate all the results is available on GitHub (https://github.com/GhedinLab/Single-Cell-IAV-infection-in-monolayer). Sequencing data that support the findings of this study have been deposited in the Gene Expression Omnibus (GEO) repository with the accession code GSE118773.
