# Supplementary figures and images for: Cell-to-Cell Variation in Defective Virus Expression and Effects on Host Responses during Influenza Virus Infection
Source: mBio. 2020 Jan 14;11(1):e02880-19. doi: 10.1128/mBio.02880-19 (PMC6960286; doi:10.1128/mBio.02880-19)

Fig S1.

(a)

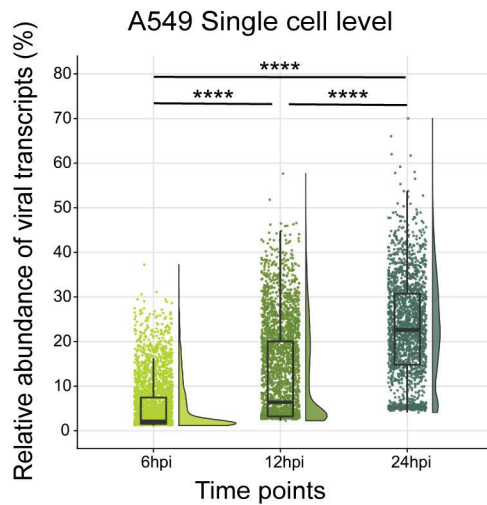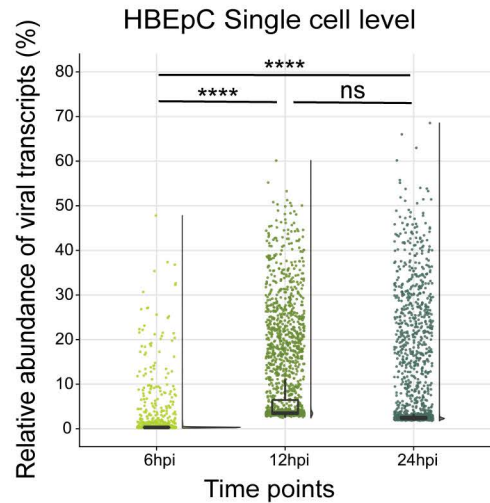

(b)

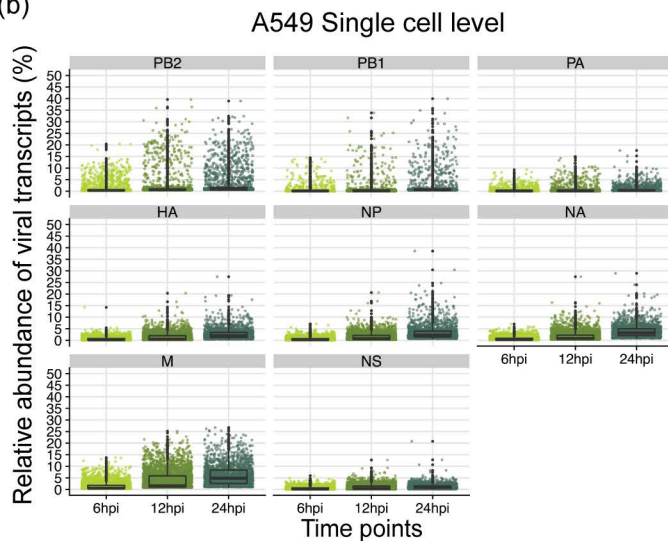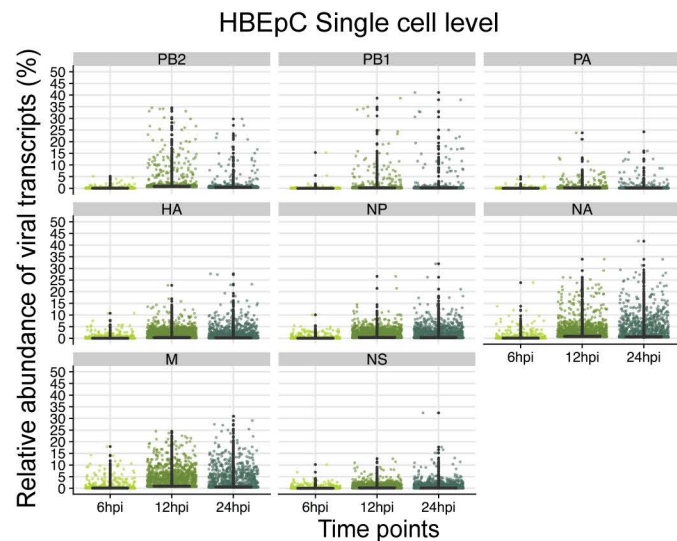

Supplement: FIG S1 [file mBio.02880-19-sf001.pdf]

Fig S2.

(a)

A549 cells

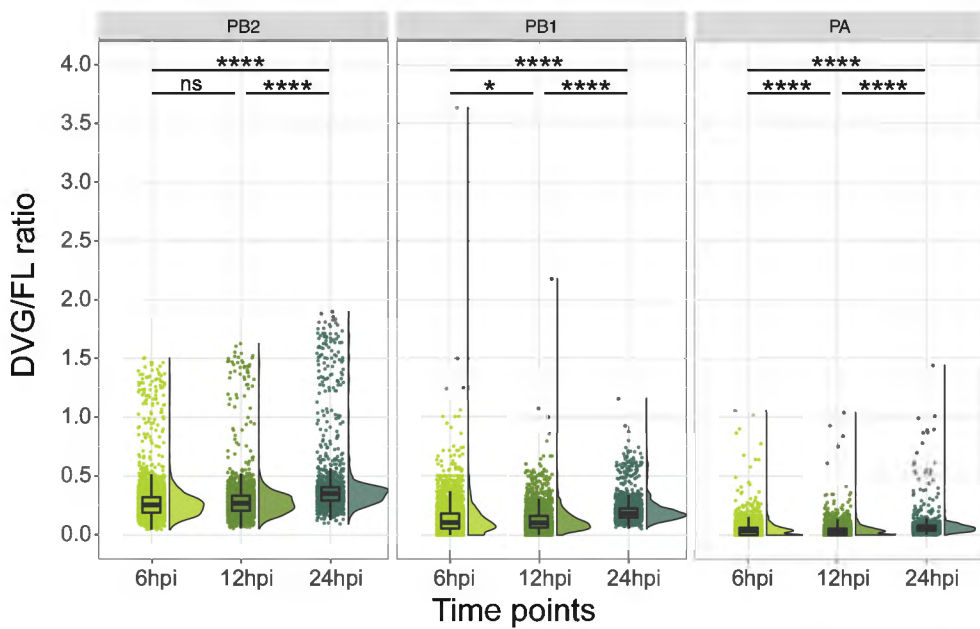

(b)

HBEpC cells

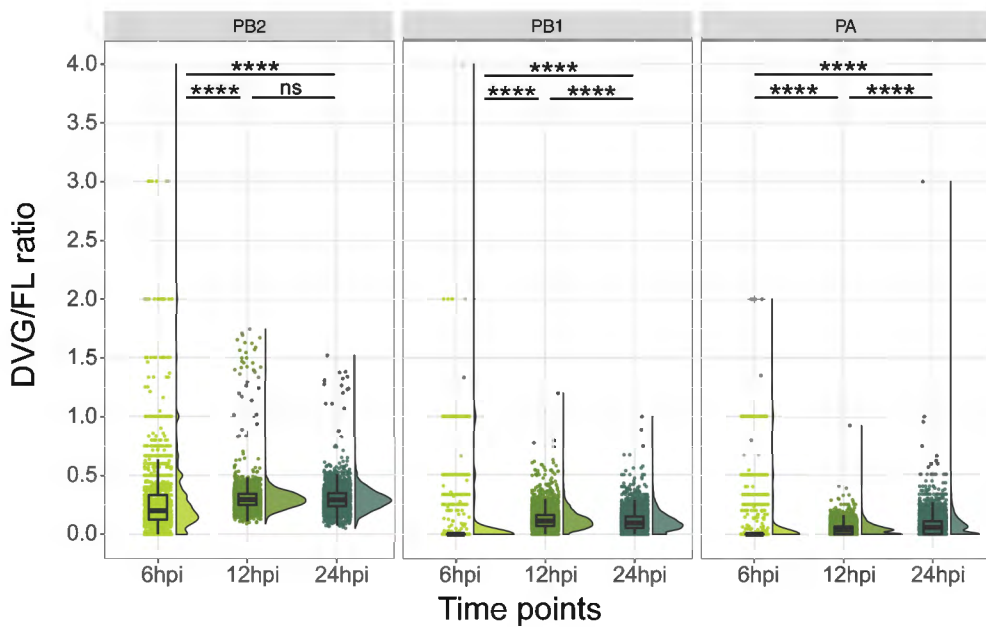

Supplement: FIG S2 [file mBio.02880-19-sf002.pdf]

Fig S3.

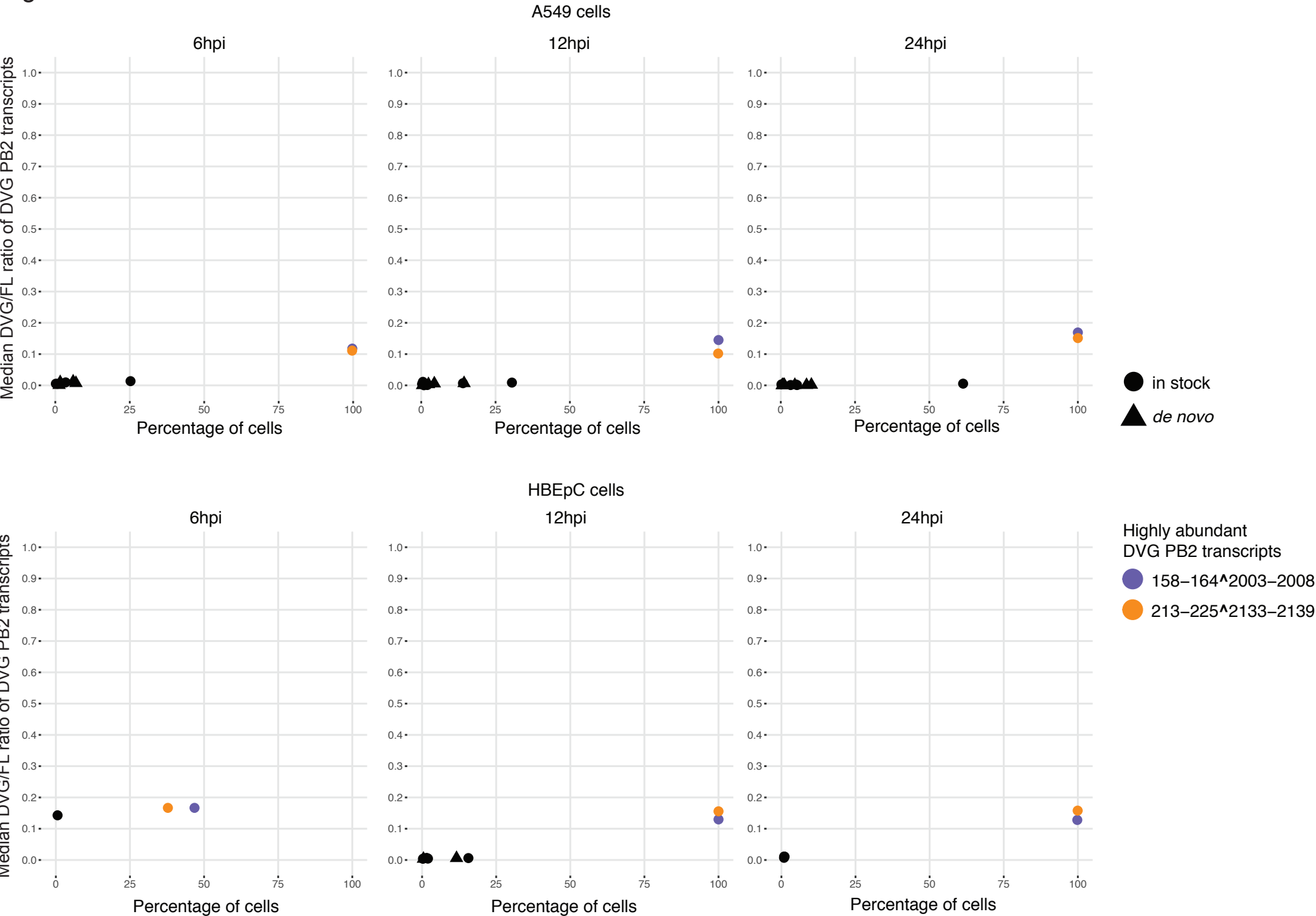

Supplement: FIG S3 [file mBio.02880-19-sf003.pdf]

Fig S4.

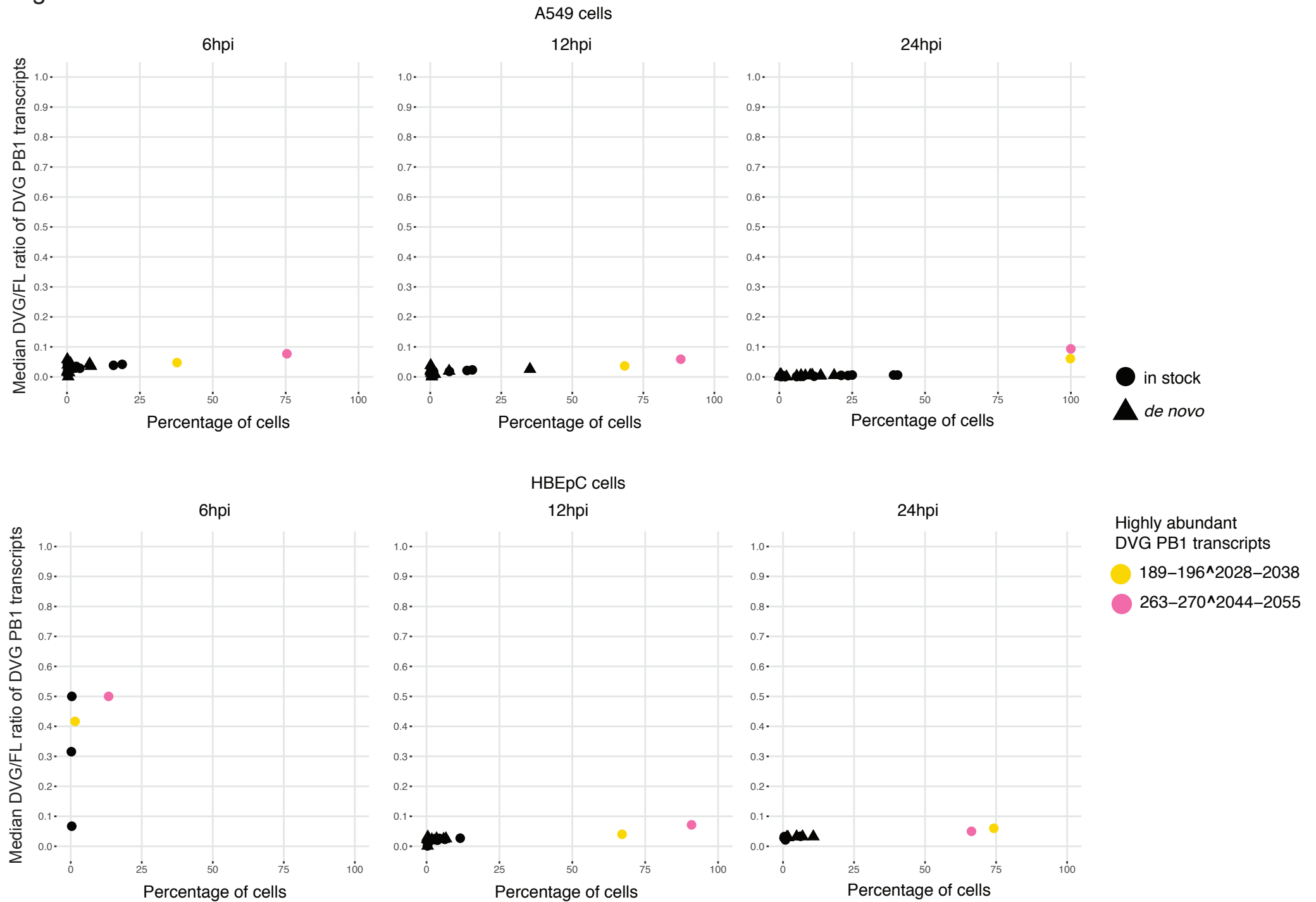

Supplement: FIG S4 [file mBio.02880-19-sf004.pdf]

Fig S5.

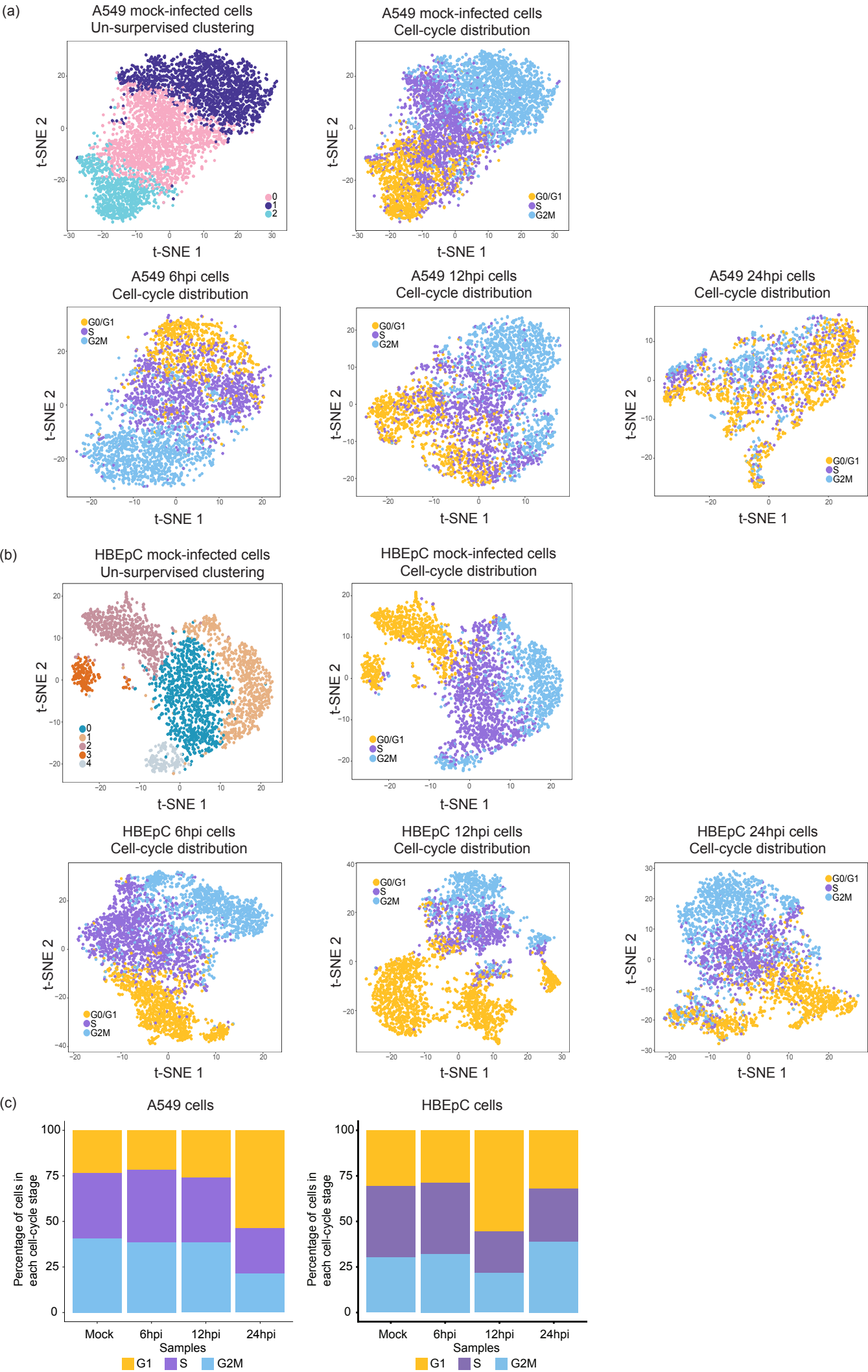

Supplement: FIG S5 [file mBio.02880-19-sf005.pdf]

Fig. S6

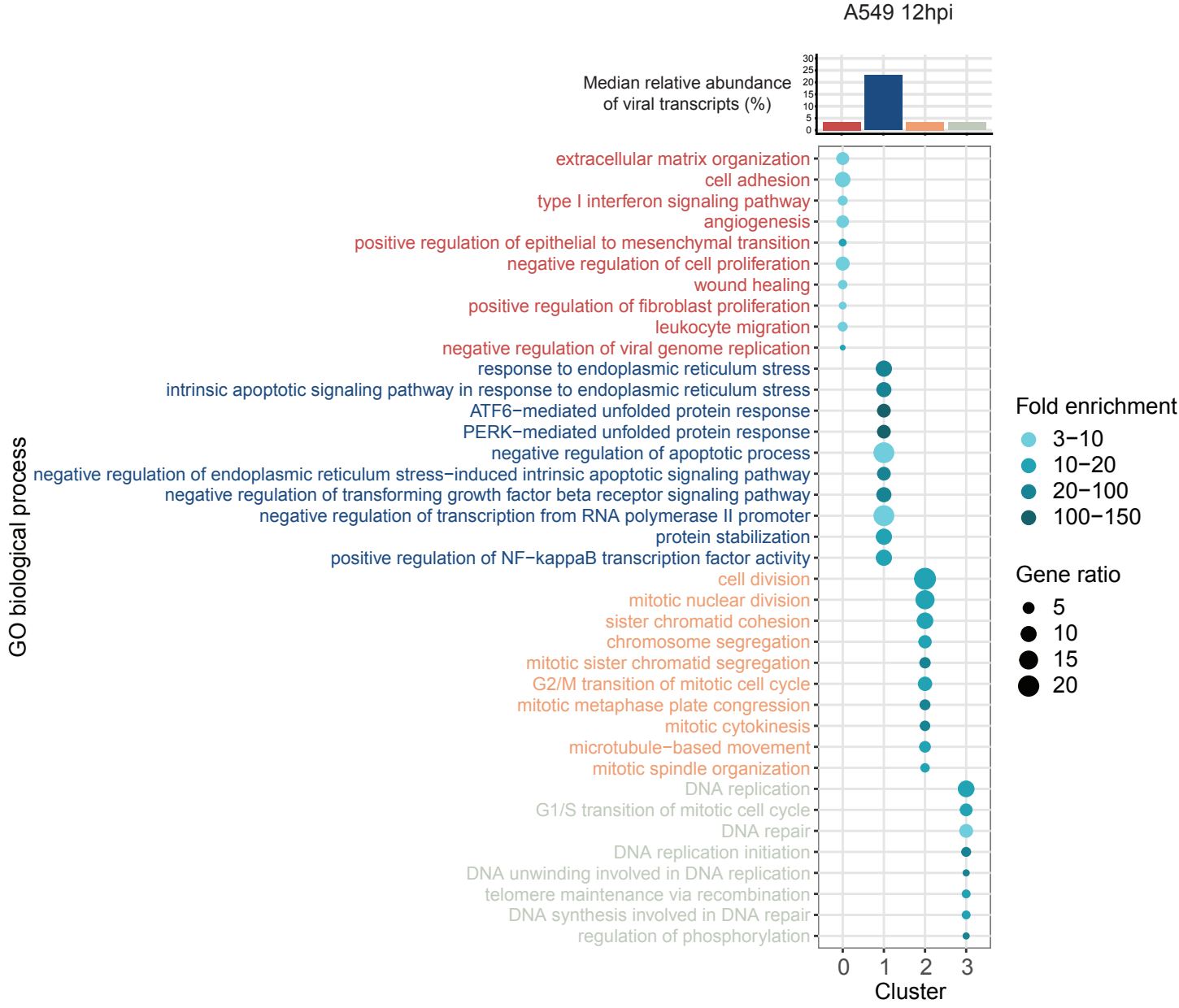

Supplement: FIG S6 [file mBio.02880-19-sf006.pdf]

Fig S7.

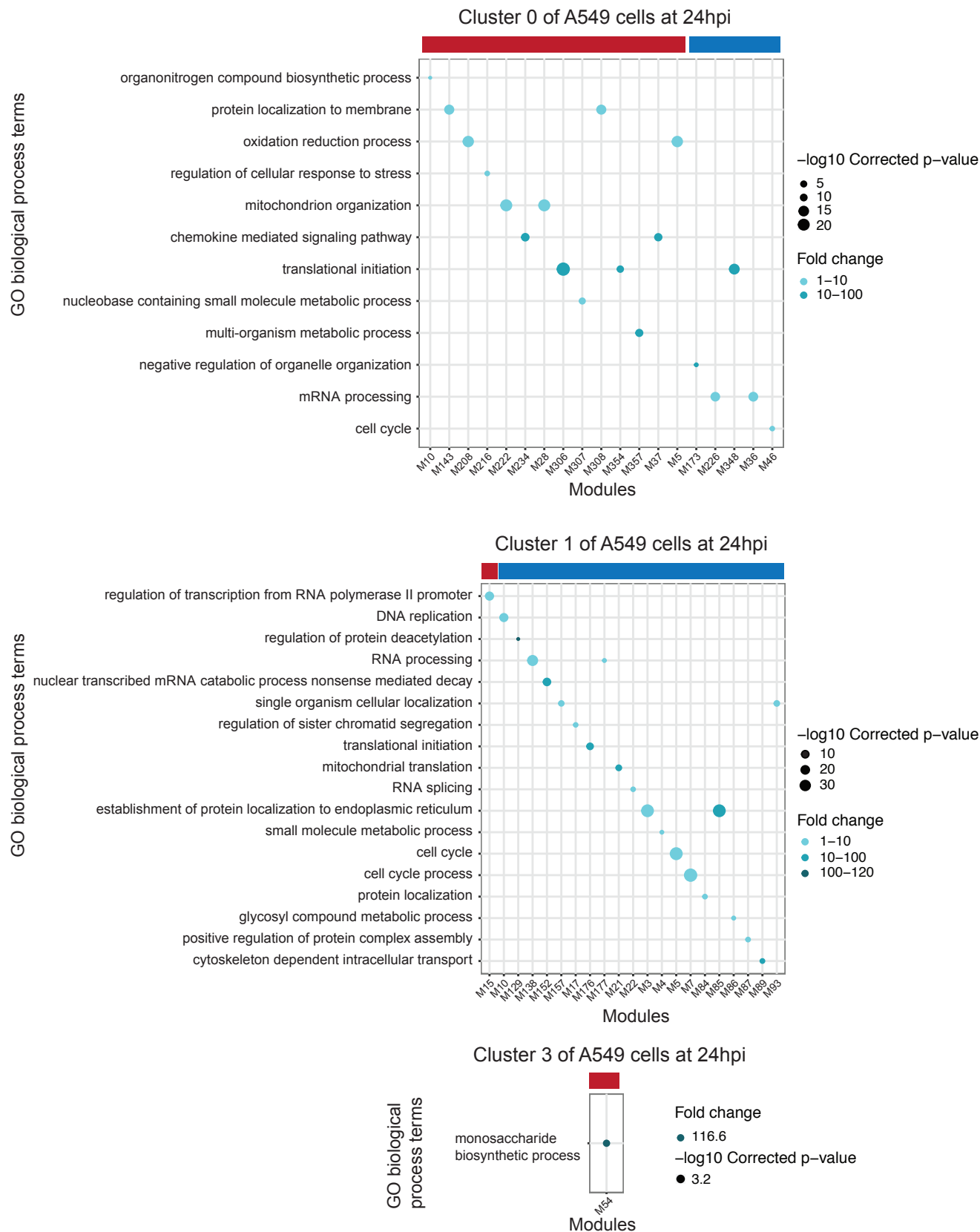

Supplement: FIG S7 [file mBio.02880-19-sf007.pdf]

Fig S8.

GO biological process terms

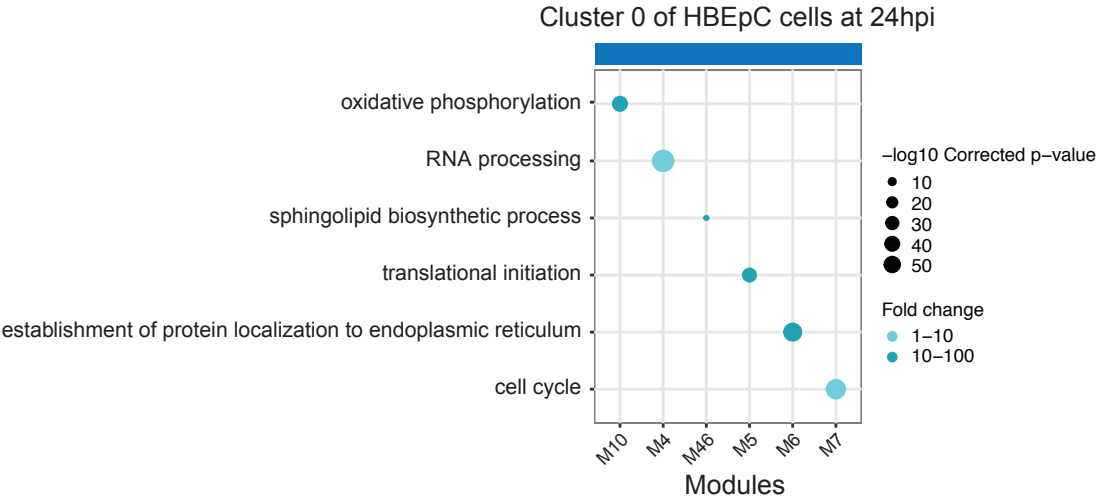

GO biological process terms

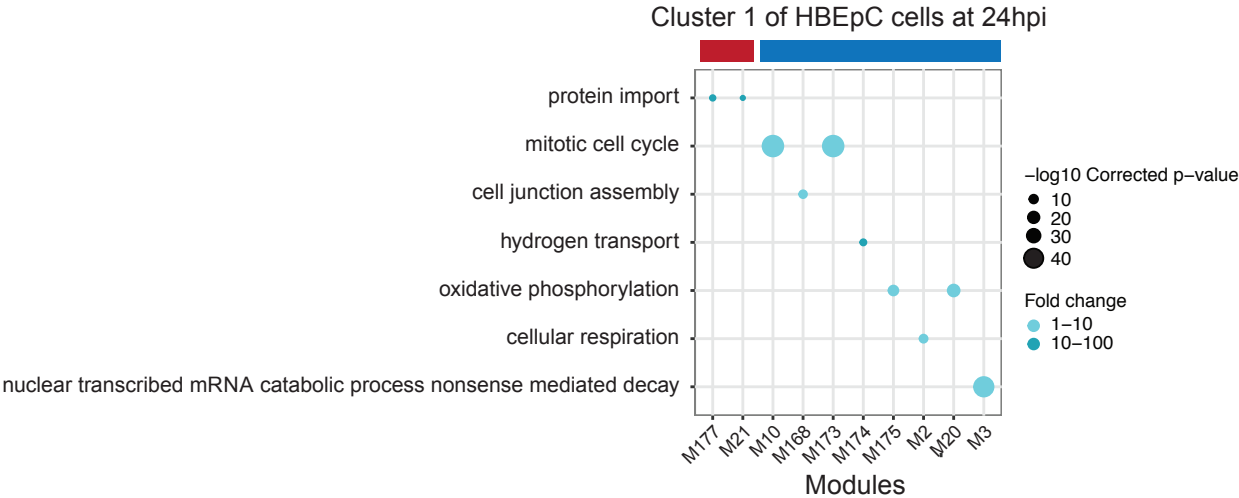

GO biological process terms

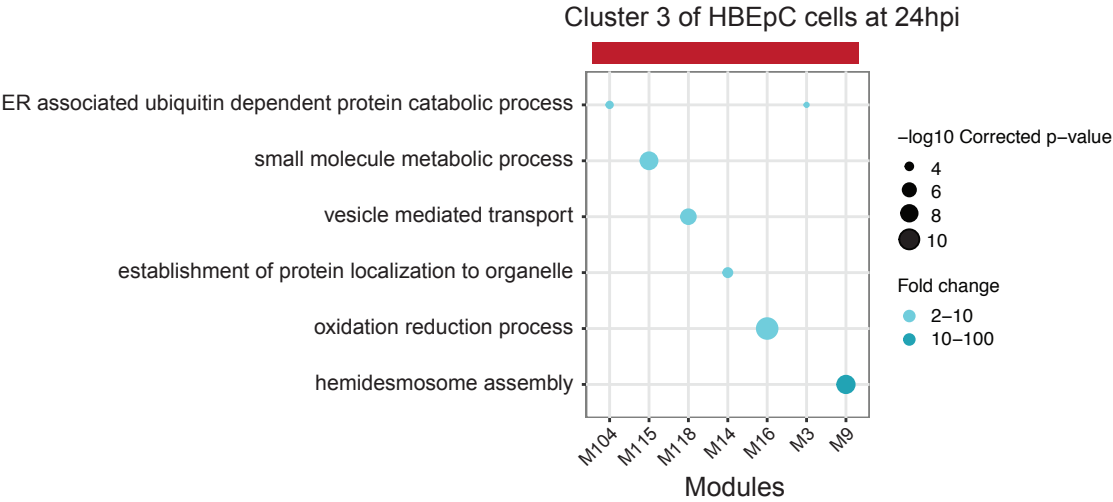

Supplement: FIG S8 [file mBio.02880-19-sf008.pdf]

Fig S9.

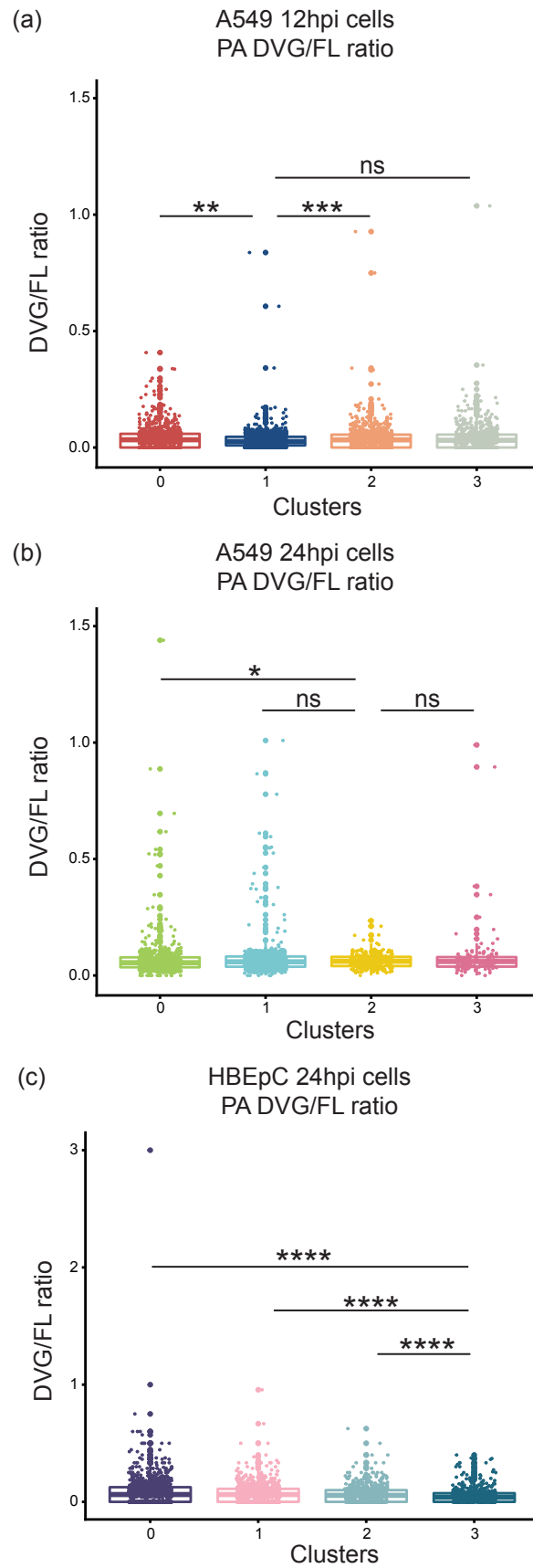

Supplement: FIG S9 [file mBio.02880-19-sf009.pdf]

Fig S10.

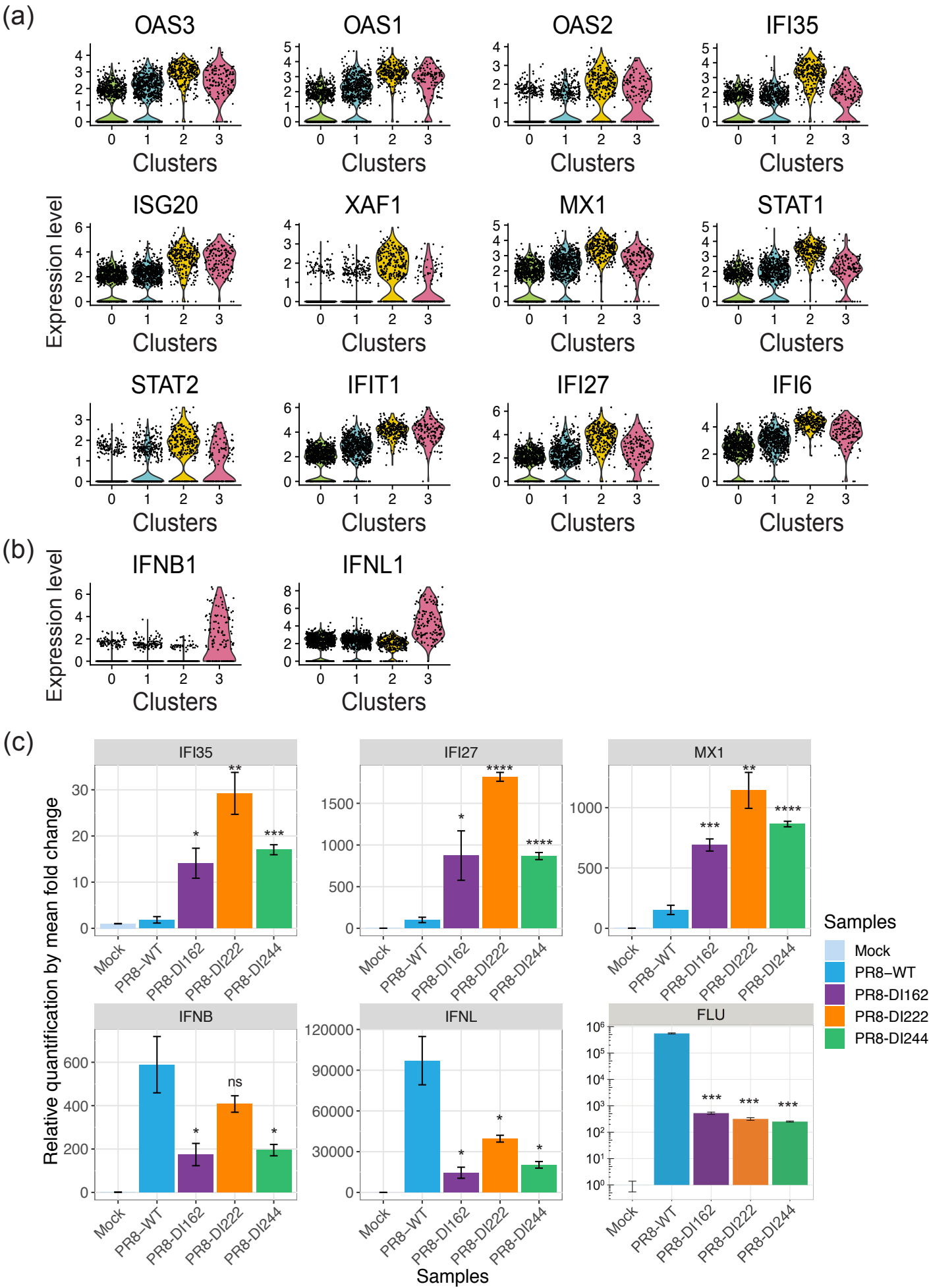

Supplement: FIG S10 [file mBio.02880-19-sf010.pdf]
